# Convergence of a linearly transformed particle method for aggregation equations

**DOI:** 10.1007/s00211-018-0958-2

**Published:** 2018-04-11

**Authors:** Martin Campos Pinto, José A. Carrillo, Frédérique Charles, Young-Pil Choi

**Affiliations:** 1 CNRS, UMR 7598, Laboratoire Jacques-Louis Lions, 75005 Paris, France; 2 Sorbonne Universités, UPMC Univ Paris 06, UMR 7598, Laboratoire Jacques-Louis Lions, 75005 Paris, France; 3 Department of Mathematics, Imperial College London, London, SW7 2AZ UK; 4 Department of Mathematics and Institute of Applied Mathematics, Inha University, Incheon, 402-751 Republic of Korea

**Keywords:** 65M12, 65M50, 82C22, 35Q70

## Abstract

We study a linearly transformed particle method for the aggregation equation with smooth or singular interaction forces. For the smooth interaction forces, we provide convergence estimates in $L^1$ and $L^\infty$ norms depending on the regularity of the initial data. Moreover, we give convergence estimates in bounded Lipschitz distance for measure valued solutions. For singular interaction forces, we establish the convergence of the error between the approximated and exact flows up to the existence time of the solutions in $L^1 \cap L^p$ norm.

## Introduction

In this work, we are interested in showing the convergence of approximated particle schemes to the Cauchy problem for the so-called aggregation equation. This equation determines the evolution of a probability density $\rho (t,x)$ defined by

1.1 $$\begin{aligned} \left\{ \begin{array}{ll} \partial _t \rho (t,x) + \nabla \cdot (\rho u)(t,x) = 0, \quad x \in \mathbb R^d, \quad t>0, &{} \\ u(t,x) = - (\nabla W * \rho (t)) (x),\quad x \in \mathbb R^d, \quad t >0, &{} \\ \rho (0,x) = \rho ^0(x)\ge 0, \quad x\in \mathbb R^d. &{} \end{array} \right. \end{aligned}$$

here $-\nabla W(x-y)$ measures the interaction force that an infinitesimal particle located at $y\in \mathbb R^d$ will exert on a particle located at $x\in \mathbb R^d$. As a result, we will call *W* the interaction potential. Since the total mass is preserved, without loss of generality, we assume

$$\begin{aligned} \int _{\mathbb R^d} \rho (t,x)\,dx =\int _{\mathbb R^d} \rho ^0(x)\,dx = 1 \qquad \forall t\ge 0. \end{aligned}$$

The microscopic dynamics of $\mathcal N$ particles $X_i$, $i=1,\dots ,\mathcal {N}$, interacting through the potential *W* are given by

1.2 $$\begin{aligned} \dot{X}_i=-\sum _{j\ne i} m_j \;\nabla W (X_i-X_j) ,\qquad i=1,\dots ,\mathcal {N}, \end{aligned}$$

where the inertia term is assumed to be negligible compared to friction [63, 64]. The macroscopic dynamics (1.1) consists of a continuity equation where the velocity field is given by $u(t,x) = -(\nabla W * \rho (t))(x)$, which is the mean-field limit of the microscopic system when $\mathcal {N}\rightarrow \infty$ under certain conditions on the potential [24, 26, 45, 53].

Equation (1.1) has attracted lots of attention in the recent years for three reasons: its gradient flow structure [2, 32, 33, 61, 73], the blow-up dynamics for fully attractive potentials [12, 14, 26, 31], and the rich variety of steady states and their bifurcations both at the discrete (1.2) and the continuous (1.1) level of descriptions [3–5, 11, 14, 22, 25, 27, 28, 49, 50, 67, 74, 75]. Furthermore, these systems are ubiquitous in mathematical modelling appearing in granular media models [10, 61], swarming models for animal collective behavior [30, 46, 59], equilibrium states for self-assembly and molecules [47, 54, 70, 76], and mean-field games in socioeconomics [17, 43] among others.

We will focus the rest of the introduction on the well-posedness of the continuous equation (1.1) and the numerical methods proposed for its approximation. Equation (1.1) has the formal structure of being a gradient flow of a functional in the set of probability measures. Indeed, defining the interaction energy as

$$\begin{aligned} \mathcal {F}[\mu ]:=\frac{1}{2} \int _{\mathbb R^d}\int _{\mathbb R^d}W(x-y)\,d\mu (x)\,d\mu (y) \end{aligned}$$

for any probability measure $\mu$, we find $u=-\nabla \frac{\delta \mathcal {F}}{\delta \mu }$ where $\frac{\delta \mathcal {F}}{\delta \mu }$ is the formal variation of the functional $\mathcal {F}[\mu ]$. This observation leads to a natural formal Lyapunov functional for the solutions of Eq. (1.1). In fact, we expect solutions to satisfy the identity

$$\begin{aligned} \frac{d}{dt} \mathcal {F}[\rho (t)]=-\int _{\mathbb R^d} |\nabla W*\rho (t)|^2 \rho (t)\,dx \end{aligned}$$

for all $t\ge 0$. This structure can be rendered fully rigorous for $C^1$-potentials [2] and it allows for mildly singular potentials at the origin [26, 27, 31] provided the interaction potential has some convexity property called $\lambda$-convexity.

On the other hand, global in time unique weak measure solutions can be constructed for any probability measure as initial data under suitable smoothness assumptions on the interaction potential. In this work, whenever we refer to *smooth potentials*, we mean that the interaction potential satisfies $\nabla W \in \mathcal {W}^{1,\infty }(\mathbb R^d)$. For smooth potentials, the approach introduced by Dobrushin for the Vlasov equation [45] using the bounded Lipschitz distance between probability measures, see [21, 24, 53] for further details, gives a well-posedness theory of weak measure solutions.

However, many of the interesting features related to blow-up dynamics and stationary states happen for potentials that are singular at the origin. Typical examples to bear in mind are combinations of repulsive attractive power-law potentials of the form $W(x)=\tfrac{|x|^a}{a}-\tfrac{|x|^b}{b}$ with $-d\le b <a$ and the convention $\tfrac{|x|^0}{0}=\log |x|$, or fully attractive potentials $W(x)=\tfrac{|x|^a}{a}$ with $a>-d$, suitably cut-off at infinity. In this work, whenever we refer to *singular potentials* we mean that the interaction potential is not smooth but satisfies

$$\begin{aligned} | \nabla W(x)|\le \frac{C}{|x|^{\alpha }} \quad \text {and} \quad | D^2 W(x)|\le \frac{C}{|x|^{1+\alpha }} \quad \text{ with } \quad - 1< \alpha < d-1 \end{aligned}$$

for some constant $C>0$, and in addition we assume that $\nabla W$ is bounded away from the origin if $\alpha < 0$. These conditions allow for singularities at the origin up to Newtonian but not including it. In particular, our singular potentials are such that $\nabla W \in \mathcal {W}^{1,q}_\mathrm{loc}(\mathbb R^d)$ with a range depending on $\alpha$: $1 \le q < \frac{d}{\alpha +1}$. Note that the power-law potentials satisfy locally the conditions of being a singular potential in the range $2-d<b < 2$ for repulsive-attractive and in the range $2-d<a < 2$ for fully attractive. The various well-posedness theories for measure solutions fail as soon as the potential becomes singular at the origin. However, weak solutions in Lebesgue spaces can be obtained. A local-in-time well-posedness theory was obtained in [15, 24] for initial data in $(L^1\cap L^{p})(\mathbb R^d)$ with $p = q'$ the conjugate exponent of *q*, and in [12, 14] a local-in-time well-posedness theory for initial data in $(L^1\cap L^\infty ) (\mathbb R^d)$ was developed for singularities up to and including a Newtonian singularity at the origin, corresponding to $\alpha = d-1$. In this work, we will use the setting introduced in [24]. The Newtonian case is very specific because of the relation between the divergence of the velocity field and the density becomes local.

Under the above assumptions of either smooth or singular potentials, the proofs of the global-in-time well-posedness of weak measure solutions and the local-in-time well-posedness of weak solutions for initial data in $(L^1 \cap L^p)(\mathbb R^d)$ spaces are essentially based on the fact that the velocity field is regular enough to have meaningful characteristics. It is proved in [15, 24, 45, 53] that the velocity field of the constructed solutions is continuous in time and Lipschitz continuous in space. Then, the flow map $\Phi _t (x)$, defined by the unique solution of the characteristic system

$$\begin{aligned} \left\{ \begin{aligned}&\frac{dX}{dt}(t)=u(t,X(t)),\\&X(s)=x, \end{aligned} \right. \end{aligned}$$

is a diffeomorphism for all times $t\ge 0$. In all cases, the solution built in [15, 24, 45, 53] is obtained by characteristics and given by $\rho (t) = \Phi _t \# \rho ^0$. Here, $\mathcal {T}\#\mu$ denotes the push-forward of a measure through a measurable map $\mathcal {T}:\mathbb R^d \longrightarrow \mathbb R^d$ defined as $\mathcal {T}\#\mu [K] := \mu [\mathcal {T}^{-1}(K)]$ for all Borel sets $K\subset \mathbb R^d$, or equivalently

$$\begin{aligned} \int _{\mathbb R^d} \varphi \, d(\mathcal {T}\#\mu ) = \int _{\mathbb R^d} (\varphi \circ \mathcal {T})\, d\mu \qquad \text{ for } \text{ all } \varphi \in C_b(\mathbb R^d). \end{aligned}$$

A very interesting question is the rigorous derivation of the continuum description (1.1) starting from the microscopic dynamics (1.2) for both regular and singular potentials. This is the so-called mean-field limit problem. The mean-field limit results contain as a by-product convergence results for the classical particle method. More precisely, proving that (1.1) is the mean-field limit of the system (1.2) as $\mathcal {N}\rightarrow \infty$ is equivalent to show that the empirical measure

$$\begin{aligned} \mu _{\mathcal {N}}(t) = \frac{1}{\mathcal {N}}\sum _{i=1}^\mathcal {N} \delta _{X_i(t)} \end{aligned}$$

converges weakly in measure sense to the solution of (1.1) provided that this weak convergence holds initially. Even if the particle method is proved to be convergent of order $\frac{1}{\mathcal {N}}$, the convergence error is only controlled in the bounded Lipschitz or Wasserstein-type distances between measures [24, 26, 45, 53].

Smooth particle methods are an extremely popular tool for the numerical simulation of a large variety of problems, mostly due to their conceptual simplicity and their automatic, mesh-free adaptation properties. They are usually referred to as Particle-In-Cell (PIC) methods in the plasma physics community where they are coupled with grid-based (Finite Difference or Finite Element) solvers for the electromagnetic field [16, 42, 48], Vortex-Blob methods for incompressible Navier–Stokes and Euler equations, see e.g. [37, 40, 62] and the references therein, and Smoothed Particle Hydrodynamics in astrophysics, see e.g. [52, 68]. More recently they have been adapted to the aggregation equation in [13] where the approximate densities are shown to converge with arbitrary orders but only in negative Sobolev norms.

This weak convergence relates to a general feature of particle methods, namely that the particle approximations to the transported density are less accurate than the approximated trajectories. On a theoretical level this is supported by the classical analysis of vortex-blob methods [7, 66] and simplified PIC schemes [39], and it is also consistent with the common observation that particle codes can provide a satisfactory description of the problem dynamics despite “noisy”, i.e. oscillating, density reconstructions.

To mitigate these oscillations, several approaches have been developed over the years. Extending the interaction radius of the smooth particles [68] (or the number of particles per cell in PIC methods [16]) is a legitimate choice that is also consistent with the standard error estimates [7, 39, 66], but leads to numerically intensive simulations. Another option is to resort to remapped or resampled particle methods where new weighted particles are periodically used to approximate the transported density [44, 60]. The resulting schemes are sometimes referred to as forward semi-Lagrangian [36, 41, 65] and their improved convergence properties can be explained by the fact that the frequent reinitializations prevent the particles to become too irregularly distributed over time. This also has a cost: reinitializing the particles can be computationally expensive, it may hamper the natural adaptivity of the particle distribution and it usually introduces numerical diffusion which may conflict with the low dissipative essence of the method.

An interesting tool is then offered by using deformed particle shapes. In these methods the particles are pushed on to discrete times according to an approximation of the exact flow as in the standard case, but each particle has its own shape which is transformed in the discrete evolution in order to better approach the local flow. Again several methods have been developed in the respective communities, and examples include transformed Gaussian shapes for plasma physics problems [6], Navier–Stokes equations [71] and astrophysics [1]. Some methods have been studied for more general transport problems, such as the spatially varying blob sizes based on appropriate mappings [8, 38, 56], the Finite Mass method [51] and the Linearly Transformed Particle (LTP) method [18]. By carefully choosing the transformation parameters as time evolves, these works obtain significant improvements in the accuracy, and mathematical proofs show that the strong convergence of the transported densities indeed holds without requiring periodic remappings or extended overlapping for the particles, see [18, 35]. In practice it has been observed that periodic remappings were still necessary to obtain satisfactory results for physically relevant problems, mostly because of the elongation of the deformed shapes, however these remappings can be done at a much lower rate than with the fixed-shape methods [18, 20].

In this article we propose and study an extension of the LTP method [18, 20] to aggregation equations. In this method each particle shape is transported by the linearized flow around its trajectory. To our knowledge the convergence of the LTP method has only been proved for a linear transport equation [18] and for a Vlasov-Poisson system [19] involving measure-preserving characteristic flows. The technical difficulties posed by the deformation of the flows in our present case have been overcome by detailed estimates of the Jacobian matrices and determinants. These estimates have allowed us to control the error on the densities via the errors of the flows to finally obtain the convergence results. Certain Sobolev regularity is needed on the initial data to obtain convergence of the LTP method in Lebesgue spaces for both smooth and singular potentials. However, a general result of convergence for weak measure solutions is obtained in an appropriate distance for measures.

We also remark that particles methods have been combined with remeshing and adaptive mesh refinement for transport and convection-diffusion equations, see [8, 9, 69] and the references therein, which also require global transforms or mapping functions related to the distortion of the flow.

Let us finally mention that other numerical methods have been proposed in the literature for the aggregation equation. In [23], the authors proposed a finite volume scheme which is shown to be energy preserving, i.e., it keeps the property that the energy functional is dissipated along the semidiscrete flow. Finite volume and finite difference schemes have been shown to be convergent to weak measure solutions of the aggregation equations for mildly singular potentials in [31, 58].

In this work, we extend the LTP method to the aggregation equation seen as one of the most important representatives of a class of nonlinear continuity equations with non divergence free velocity fields in any dimensions. We start by summarizing the basic ideas of the numerical LTP method in Sect. 2 together with the preliminaries and notations used in this work. Section 3 is devoted to give convergence results for smooth potentials in Lebesgue spaces. Depending on the regularity of the initial data, we will be able for smooth potentials to control errors in $L^1$ and $L^\infty$. For initial data just being a probability measure, we will show in Sect. 4 the convergence in bounded Lipschitz distance. In the case of singular potentials, we will control in Sect. 5 the error up to the existence time of the solution of (1.1) in $L^1$ and $L^p$ with *p* suitably chosen. We finally show in Sect. 6 the performance of this method in one dimension validating the numerical implementation with explicit solutions and making use of it to study certain not well-known qualitative features of the evolution of (1.1) with several smooth and singular potentials.

## Preliminaries

### Basic properties of the exact flow

In the setting of our main results, the velocity field of the exact solution to (1.1) is always continuous in *t* and Lipschitz continuous in *x*. The solution of the characteristic system

$$\begin{aligned} \left\{ \begin{aligned}&\frac{dX(t)}{dt}=u(t,X(t)),\\&X(s)=x, \end{aligned} \right. \end{aligned}$$

is well-defined and it has unique global in time solutions for all initial data $x\in \mathbb R^d$. Moreover, the general solution of the characteristic system is a diffeomorphism in $\mathbb R^d$. The general flow map will be denoted by $F^{s,t}(x)$ for all $t,s\in \mathbb R$ and $x\in \mathbb R^d$.

As discussed in the introduction, the solutions to (1.1) can always be expressed as $\rho (t) = F^{0,t} \# \rho ^0$ or equivalently as

$$\begin{aligned} \rho (t,x)=\rho ^0\left( F^{t,0}(x)\right) j^{t,0}(x) \quad \text {with} \!\quad \! j^{t,0}(x) = \det ( J^{t,0}(x)),\quad J^{t,0}(x) = D F^{t,0}(x). \end{aligned}$$

The flow map satisfies

2.1 $$\begin{aligned} F^{s,t}(x) = x + \int _s^t u(\tau ,F^{s,\tau }(x))d\tau = x - \int _s^t (\nabla W * \rho (\tau ))(F^{s,\tau }(x))d\tau , \end{aligned}$$

and the Jacobian matrix and its determinant satisfy the differential equations

2.2 $$\begin{aligned} \frac{d}{dt}J^{s,t}(x) = D u(t,F^{s,t}(x)) J^{s,t}(x) \quad \text{ and } \quad \frac{d}{dt}j^{s,t}(x) = \nabla \cdot u(t,F^{s,t}(x)) j^{s,t}(x). \end{aligned}$$

Using $u(\tau ,y) = -(\nabla W * \rho (\tau ))(y)$, this yields

2.3 $$\begin{aligned} J^{s,t}(x) - I_{d}&= \int _s^t \! D u(\tau , F^{s,\tau }(x)) J^{s,\tau }(x)d\tau \nonumber \\&= - \int _s^t \! (D^2 W * \rho (\tau ))(F^{s,\tau }(x)) J^{s,\tau }(x)d\tau \end{aligned}$$

and

2.4 $$\begin{aligned} j^{s,t}(x)&= \exp \left( -\int _s^t \nabla \cdot u(\tau ,F^{s,\tau }(x) d\tau \right) \nonumber \\&= \exp \left( -\int _s^t (\Delta _x W * \rho (\tau ))(F^{s,\tau }(x)) d\tau \right). \end{aligned}$$

Estimates are then easily derived when $u \in L^\infty (0,\infty ;\mathcal {W}^{1,\infty }(\mathbb R^d))$. We will write $L:=\sup _{t\in [0,\infty )} \Vert u(t,\cdot )\Vert _{\mathcal {W}^{1,\infty }}$. For instance, using (2.2) and $J^{s,s}(x) = I_d$ we find

2.5 $$\begin{aligned} \sup _{x \in \mathbb R^d} |J^{s,t}(x)| \le \exp \left( C L |t-s|\right), \end{aligned}$$

and in particular the characteristic flow is Lipschitz (relative to any norm in $\mathbb R^d$),

2.6 $$\begin{aligned} |F^{s,t}|_{Lip} \le \exp \left( C L |t-s|\right). \end{aligned}$$

Furthermore, we derive from (2.3) and (2.5) that

2.7 $$\begin{aligned} \sup _{x \in \mathbb R^d} |I_{d} - J^{s,t}(x)| \le (t-s)\exp \left( C L |t-s|\right) \end{aligned}$$

and using (2.4) we also find

2.8 $$\begin{aligned} \exp \left(-C L |t-s|\right) \le j^{s,t}(x) \le \exp \left(C L |t-s|\right) \quad \text { for } \quad x \in \mathbb R^d \end{aligned}$$

and

2.9 $$\begin{aligned} \Vert j^{s,t}-1\Vert _{L^\infty } \le C L |t-s| \exp \left(C L(t-s)\right). \end{aligned}$$

Let us remark that the previous estimates (2.5)–(2.9) can also be obtained in a time interval [0, *T*] for locally Lipschitz velocity fields $u \in L^\infty (0,T;\mathcal {W}^{1,\infty }(\mathbb R^d))$ for some $T>0$, with constant $L_T:=\sup _{t\in [0,T]} \Vert u(t,\cdot )\Vert _{\mathcal {W}^{1,\infty }}$. These estimates will be used in Sect. 5, where the dependence on T of the Lipschitz constant will be omitted for the sake of simplicity.

### Linearly transformed particles

As in standard particle methods, the density $\rho$ is represented with weighted macro-particles, and as in smooth particle methods, particles have here a finite and smooth shape. Thus, we approximate the initial density $\rho ^0$ on a Cartesian grid of size $h > 0$ by

2.10 $$\begin{aligned} \rho _{h}^0(x)=\sum _{k\in \mathbb Z^d} \omega _k \varphi _{h,k}^0(x) \end{aligned}$$

with particle shapes obtained by scaling and translating a reference function, i.e.

2.11 $$\begin{aligned} \varphi _{h,k}^0(x)=\frac{1}{h^d} \varphi \left( \frac{x-x_k^0}{h}\right) , \qquad x_k^0=kh. \end{aligned}$$

Here the reference shape is assumed to have a compact support ${{\mathrm{supp}}}(\varphi ) \subset B(0,R_o)$, be bounded and satisfy

$$\begin{aligned} \sum _{k\in \mathbb Z^d} \varphi (x - k) = 1 \quad \text {for }x \in \mathbb R^d \qquad \text { and } \qquad \int _{\mathbb R^d} \varphi =1. \end{aligned}$$

In this work we will require that the shape functions are Lipschitz, and we can either consider for the reference shape the tensor-product hat function

2.12 $$\begin{aligned} \varphi (x) = \prod _{1\le i\le d} \max (1-|x_i|,0). \end{aligned}$$

or the B3-spline

2.13 $$\begin{aligned} \varphi (x)= \frac{1}{6}\left\{ \begin{array}{ll} \left( 2-|x|\right) ^3 &{}\quad \text {if }1 \le |x|< 2, \\ \displaystyle 4-6x^2 +3 |x|^3 &{}\quad \text {if } 0\le |x| < 1, \\ 0 &{} \quad \text {otherwise.} \end{array} \right. \end{aligned}$$

As for the weights $\omega _k = \omega _k(h,\rho ^0)$, they are usually defined as

2.14 $$\begin{aligned} \omega _k = \int _{x_k^0 + \left[-\frac{h}{2}, \frac{h}{2}\right]^d} \rho ^0(x) dx, \end{aligned}$$

however this will not be sufficient to prove the convergence of our particle scheme without additional smoothness assumptions on the initial density $\rho ^0$. Indeed, using standard arguments (see e.g. [18, 34]) based on the fact that the approximation $\rho ^0 \mapsto \rho ^0_h = \sum _{k\in \mathbb Z^d} \omega _k \varphi _{h,k}^0$ is local, bounded in any $L^p$ space and preserves the affine functions, one easily verifies the following estimate.

#### Proposition 1

If $\rho ^0_h$ is initialized as in (2.10) with weights and shape function given by (2.14) and (2.11), respectively, then we have

2.15 $$\begin{aligned} ||\rho ^0-\rho ^0_h||_{L^p} \le C h^s ||\rho ^0||_{\mathcal {W}^{s,p}} \end{aligned}$$

for $s \in \{0, 1, 2\}$, $1\le p \le \infty$ and a constant *C* independent of $\rho ^0$.

In our analysis we will need second-order estimates which are then available for $\rho ^0 \in \mathcal {W}^{2,p}(\mathbb R^d)$. However, if we allow negative weights then second-order estimates are also available in a dual norm, as follows. Consider weights defined as

2.16 $$\begin{aligned} \omega _k = \int _{\mathbb R^d} {\tilde{\varphi }}_{h,k}^0(x) \rho ^0(x)dx \end{aligned}$$

with integration kernels bi-orthogonal to the shape functions in the sense that

2.17 $$\begin{aligned} \int _{\mathbb R^d} \varphi _{h,k}^0 {\tilde{\varphi }}_{h,k'}^0 = \delta _{k,k'} \end{aligned}$$

holds with $\delta _{k,k'}$ the Kronecker symbol. Similar to the shape functions, they can be obtained by scaling and translating a reference ${\tilde{\varphi }}$ (assumed again compactly supported, bounded and satisfying (2.12)) with a different normalization, namely

2.18 $$\begin{aligned} {\tilde{\varphi }}_{h,k}^0(x)=\tilde{\varphi }\left( \frac{x-x_k^0}{h}\right) . \end{aligned}$$

**Fig. 1:**
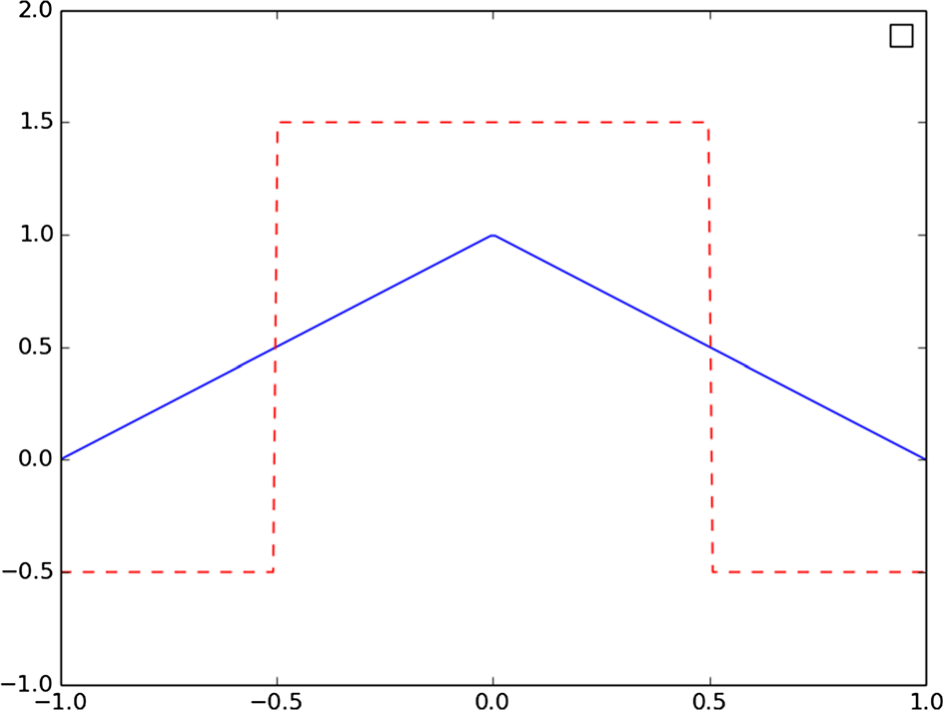
A piecewise affine shape function and its bi-orthogonal kernel (dotted line). Both functions vanish outside $[-1,1]$

For instance if $\varphi$ is the above tensor-product hat function (2.12) then for the integration kernel we may take ${\tilde{\varphi }}(x) = \prod _{i\le d} \big (\frac{3}{2} {\mathbb {1}}_{[-\frac{1}{2},\frac{1}{2}]} - \tfrac{1}{2} {\mathbb {1}}_{[-1,-\frac{1}{2}] \cup [\frac{1}{2},1]}\big )(x_i),$ see Fig. 1.

Notice that estimate (2.15) still holds with these weights. Now, from the duality (2.17) we can derive a convenient second-order estimate which only relies on the first-order smoothness of $\rho ^0$. It is expressed in the dual norm

$$\begin{aligned} \displaystyle ||w||_{\mathcal {W}^{-1,p}} := \sup _{v \in \mathcal {W}^{1,q}(\mathbb R^d)} \langle w,v\rangle / ||v||_{\mathcal {W}^{1,q}}, \end{aligned}$$

where *q* is the conjugate exponent of *p* and $\langle w,v\rangle$ is the duality pair that coincides with the integral of the product *wv* as soon as the latter is integrable.

#### Proposition 2

If $\rho ^0_h$ is initialized as in (2.10) with shape functions and weights satisfying properties (2.11) and (2.12) and (2.16)–(2.18), we have

2.19 $$\begin{aligned} ||\rho ^0-\rho ^0_h||_{\mathcal {W}^{-1,p}} \le C h^2 ||\rho ^0||_{\mathcal {W}^{1,p}} \end{aligned}$$

for $1 \le p \le \infty$, with a constant *C* independent of *h*.

#### Proof

It follows from the duality relation (2.17) that $\langle \rho ^0 - \rho ^0_h,{\tilde{\varphi }}_{h,k}^0\rangle = 0$ for all *k*. In particular, given $v \in \mathcal {W}^{1,\infty }(\mathbb R^d)$ we have

$$\begin{aligned} \langle \rho ^0 - \rho ^0_h,v\rangle = \langle \rho ^0 - \rho ^0_h,v - \tilde{v}_h\rangle \end{aligned}$$

with $\tilde{v}_h := \sum _{k \in \mathbb Z^d} \langle v,\varphi _{h,k}^0\rangle {\tilde{\varphi }}_{h,k}^0$ and standard arguments show that the approximation $v \mapsto \tilde{v}_h$ satisfies an error estimate similar to (2.15) for $s=1$. Using the Hölder inequality this gives

$$\begin{aligned} \langle \rho ^0 - \rho ^0_h,v\rangle \le ||\rho ^0 - \rho ^0_h||_{L^p} ||v - \tilde{v}_h||_{L^q} \le C h^2 ||\rho ^0||_{\mathcal {W}^{1,p}}||v||_{\mathcal {W}^{1,q}} \end{aligned}$$

and the proof is completed due to the definition of the $\mathcal {W}^{-1,p}(\mathbb R^d)$ norm. $\square$

We observe that both the above initializations yield

2.20 $$\begin{aligned} \sup _{k\in \mathbb Z^d} |\omega _k| \le C h^{d/q} \Vert \rho ^0\Vert _{L^{p}} \qquad \text { where } \qquad \tfrac{1}{q} + \tfrac{1}{p} = 1, \end{aligned}$$

and since the shape functions are assumed to be non-negative, (2.12) gives

2.21 $$\begin{aligned} \Vert \rho _h^0\Vert _{L^1} \le \sum _{k\in \mathbb Z^d} |\omega _k| \le C \Vert \rho ^0 \Vert _{L^1} \le C, \end{aligned}$$

with a constant depending only on ${\tilde{\varphi }}$.

We now describe the LTP method. As mentioned in the introduction, compared to standard particle methods, the LTP method follows the shape of smooth particles. Therefore we need to track not only the particle positions but also their deformations given by the Jacobian matrices. Given discrete trajectories $x^n_k$ approximating the exact ones $F^{0,t_n}(x^0_k)$ on the discrete times

$$\begin{aligned} t_n := n\Delta t, \qquad n = 0, 1, \ldots , N := T / \Delta t, \end{aligned}$$

and non singular approximations $J^n_k$ of the forward Jacobian matrices $J^{t_n,t_{n+1}}(x^n_k)$, the particle shapes $\varphi ^{n+1}_{h,k}$ are recursively defined as the push-forward of $\varphi ^{n}_{h,k}$ along the affine flow

2.22 $$\begin{aligned} F_{h,k}^{n}: \, x \mapsto x_k^{n+1} + J_{k}^{n} (x-x_k^{n}), \end{aligned}$$

which approximates the exact flow $F^{t_{n},t_{n+1}}$ around $x_k^{n}$. Here $x_k^{n+1}$ can also be seen as an approximation to $F^{t_{n},t_{n+1}}(x_k^{n})$, as will be specified below. In short, we define

$$\begin{aligned} \varphi ^{n+1}_{h,k} := F_{h,k}^{n} \# \varphi ^{n}_{h,k} = \frac{1}{j_k^{n}} \varphi ^{n}_{h,k}\circ (F_{h,k}^{n})^{-1}, \end{aligned}$$

where $j_k^{n}:=\det (J_k^n) > 0$. Starting from $\varphi ^{0}_{h,k}$ defined as in (2.11), this gives particles of the form

2.23 $$\begin{aligned} \varphi _{h,k}^n(x) := \frac{1}{h_k^n} \varphi \left( \frac{D_k^n (x- x_k^n)}{h} \right) , \end{aligned}$$

where the deformation matrix $D_k^{n}$ and the particle volume $h_k^{n}$ are defined by

2.24 $$\begin{aligned} {\left\{ \begin{array}{ll} D_k^{n+1} := D_k^{n} \left( J_k^{n}\right) ^{-1} \\ h_k^{n+1} := j_{k}^{n} h_k^{n} = \det (J_k^{n}) h_k^{n} \end{array}\right. } \text { with } \quad {\left\{ \begin{array}{ll} D_k^{0} := I_{d} \\ h_k^{0} := h^d \end{array}\right. }. \end{aligned}$$

It follows from the above process that $D_k^n$ is an approximation to the backward Jacobian matrix $J^{t_n,0}(x^n_k)$, whereas $h_k^n$ approximates the elementary volume $h^d$ multiplied by the Jacobian determinant of the forward flow $F^{0,t_n}$ at $x^0_k$. Moreover, the particle shape $\varphi _{h,k}^n$ is the push-forward of $\varphi ^0_{h,k}$ along the integrated flow

2.25 $$\begin{aligned} \overline{F}_{h,k}^n := F^{n-1}_{h,k} \circ \cdots \circ F^{0}_{h,k} : x \mapsto x^n_k + \overline{J}^n_k (x-x^0_k) \quad \text { where } \quad \overline{J}^n_k := (D^n_k)^{-1} \end{aligned}$$

which can be seen as a linearization of $F^{0,t_n}$ around $x^0_k$ (for $n=0$ we set $\overline{F}_{h,k}^0 = I$ since $D^0_k = I_d$). Indeed, it follows from the above definitions that

2.26 $$\begin{aligned} \varphi _{h,k}^n = \overline{F}_{h,k}^{n} \# \varphi ^{0}_{h,k}, \end{aligned}$$

and we easily verify that

$$\begin{aligned} h_k^n = h^d \det (J_{h,k}^{n-1}) \, \cdots \, \det (J_{h,k}^0) = \frac{h^d}{\det (D_{h,k}^n)} \approx h^d \det (J^{0,t_n}(x^0_k)). \end{aligned}$$

Finally, the LTP approximation of the density at time $t_n$ is defined as

2.27 $$\begin{aligned} \rho _{h}^n(x) := \sum _{k\in \mathbb Z^d} \omega _k \varphi _{h,k}^n(x) \end{aligned}$$

with weights $\omega _k$ constant in time and computed as in (2.14) or (2.16). According to (2.26), we have $\int \varphi ^{n}_{h,k} =\int \varphi _{h,k}^0 =\int \varphi$, and thus the conservation of mass ($\int \rho _{h}^n = \int \rho _{h}^0$) holds at the discrete level. Moreover, using the fact that the particle shapes are non-negative, we find as in (2.21)

2.28 $$\begin{aligned} \Vert \rho _h^n\Vert _{L^1} \le \sum _{k\in \mathbb Z^d} ||\omega _k \varphi ^{n}_{h,k}||_{L^1} = \sum _{k\in \mathbb Z^d} |\omega _k| \le C \Vert \rho ^0 \Vert _{L^1} = C, \qquad n\ge 0. \end{aligned}$$

### Approximated Jacobian matrices and particle positions

To complete the description of the numerical method (2.23) and (2.24), (2.27), we are left to specify how to compute the particle center $x^{n+1}_k$ and the discrete Jacobian matrix $J^n_k$ involved in the affine flow (2.22). Before doing so we observe that if the matrices $D^2W(x)$ and $D^2W(y)$ commute for all *x* and *y*, then the exact solution to the ODE (2.2) takes an exponential form. However, in the general case the matrix $J^{t_n,t_{n+1}}(x)$ is *not* equal to

2.29 $$\begin{aligned} \tilde{J}^{t_n,t_{n+1}}(x) := \exp \left( -\int _{t_n}^{t_{n+1}} (D^2 W * \rho (\tau ))(F^{t_n,\tau }(x))d\tau \right) \end{aligned}$$

but the difference is small, as shown next.

#### Proposition 3

If $u \in L^{\infty }(0,T;\mathcal {W}^{1,\infty }(\mathbb R^d))$, then we have

$$\begin{aligned} |\tilde{J}^{t_n,t_{n+1}}(x) - J^{t_n,t_{n+1}}(x) | \le C (\Delta t)^2 \quad \text { for } \quad x \in \mathbb R^d, \end{aligned}$$

with a constant *C* independent of $n \le N-1$ and $\Delta t$.

#### Proof

Given $n \le N-1$ and $x \in \mathbb R^d$, we denote for simplicity

$$\begin{aligned} B(\tau ) = B(\tau , t_n, x) := (D^2 W * \rho (\tau ))(F^{t_n,\tau }(x)) \end{aligned}$$

and we observe that $|B(\tau )| \le L = \sup _{t\le T} |u(t)|_{\mathcal {W}^{1,\infty }}$ for all $\tau \in [t_n,t_{n+1}]$. From (2.3) we have $J^{t_n,t_{n+1}}(x) = I_d - \int _{t_n}^{t_{n+1}} B(\tau ) d\tau + \int _{t_n}^{t_{n+1}} B(\tau )(I_d - J^{t_n,\tau }(x)) d\tau$, hence the difference $E(x) := \tilde{J}^{t_n,t_{n+1}}(x) - J^{t_n,t_{n+1}}(x)$ can be decomposed into

$$\begin{aligned} E(x) = \underbrace{\sum _{m=2}^\infty \frac{(-1)^m}{m !} \left( \int _{t_n}^{t_{n+1}} B(\tau ) d\tau \right)^m}_{=: (a)} + \underbrace{\int _{t_n}^{t_{n+1}} B(\tau ) (I_{d} - J^{t_n,\tau }(x))d\tau }_{=: (b)}. \end{aligned}$$

From the above bound for *B* we readily find $(a) \le \sum _{m=2}^\infty \frac{1}{m !}(C\Delta t)^m \le C(\Delta t)^2$. Turning to (*b*), we use again (2.3) to write

$$\begin{aligned} |(b)| = \left| \int _{t_n}^{t_{n+1}} B(\tau ) \left( \int _{t_n}^\tau B(t) J^{t_n,t}(x) dt \right) d\tau \right| \le C \int _{t_n}^{t_{n+1}} \int _{t_n}^\tau |J^{t_n,t}(x)| dt d\tau \le C (\Delta t)^2 \end{aligned}$$

where we have used (2.5) in the last inequality. The result follows. $\square$

At time $t_{n+1}$, $x_k^{n+1}$ is an approximation of $F^{t_{n},t_{n+1}} (x_k^{n})$ which is the solution at time $t_{n+1}$ of the ODE

2.30 $$\begin{aligned} \left\{ \begin{aligned}&\frac{d\tilde{X}_{k}(t)}{dt}= u(t,\tilde{X}_{k}(t)) = - (\nabla W * \rho (t))(\tilde{X}_{k}(t)),\\&\tilde{X}_k(t_n)=x_k^n. \end{aligned} \right. \end{aligned}$$

Then we can define $x_k^{n+1}$ as the approximation given by a numerical scheme discretizing (2.30) when replacing the exact density $\rho$ at discrete times in $[t_n,t_{n+1}]$ by its LTP approximation $\rho _h^n$. In the convergence analysis, we consider particle trajectories $x_k^n$ and approached Jacobian matrices $J_k^n$ defined by an explicit Euler scheme:

2.31 $$\begin{aligned} \left\{ \begin{aligned}&x_k^{n+1} :=x_k^n -\Delta t \,(\nabla W * \rho _h^n)(x_k^n),\\&J_k^{n} := e^{- \Delta t \,(D^2W * \rho _h^n)(x_k^n)} =\sum _{m=0}^\infty \frac{(-1)^m}{m !} \left[ \Delta t \, ((D^2 W * \rho ^n_h)(x_k^n)\right] ^m. \end{aligned} \right. \end{aligned}$$

Note that this expression can be seen as an approximation to (2.29) using a rectangular rule in the time integral (here will not take into account the approximation error of convolution products). Accordingly, we set

2.32 $$\begin{aligned} j_k^n = \det (J_k^n) = \exp \left( -\Delta t (\Delta _x W * \rho ^n_h)(x_k^n)\right). \end{aligned}$$

Using (3.1) and the $L^1$ bound (2.28) on $\rho ^n_h$, we see that this approximation yields

$$\begin{aligned} | J_k^n - I_d | = \left| \sum _{m=1}^\infty \frac{(-1)^m}{m !} (\Delta t)^m ((D^2 W * \rho ^n_h)(x_k^n))^m\right| \le \sum _{m=1}^\infty \frac{1}{m !}(C\Delta t)^m \le C\Delta t e^{C \Delta t}. \end{aligned}$$

We note that higher-order time discretizations are also possible. To avoid extensive technicalities we will not consider them here, as this article focuses on the space discretization.

#### Remark 1

When $d>1$, computing the exponential of a $d \times d$ matrix is costly. Another possibility is to approximate $J^{t_n,t_{n+1}}(x_k^n)$ by

$$\begin{aligned} \tilde{J_k^n} = I_{d} - \Delta t \, (D^2W * \rho ^n_h)(x_k^n). \end{aligned}$$

It is easily verified that the difference between these approximations satisfies

$$\begin{aligned} \sup _{0\le n\le \frac{T}{\Delta t}} \sup _{k\in \mathbb Z} \left\| \tilde{J_k^n} - J_k^n \right\| = {\mathcal O} \left( {\Delta t^2} \right) \end{aligned}$$

as long as we have $\nabla W\in \mathcal {W}^{1,q}(\mathbb R^d)$ and $\sup _{0\le n\le \frac{T}{\Delta t}} \Vert \rho _h^n \Vert _{L^p} \le C$ with $p = q'$.

### General strategy of the convergence proofs

In order to establish error estimates for the approximation of the density $\rho (t_n)$ by $\rho ^n_h$ we will use Gronwall arguments that involve errors on the flows and on the Jacobian determinants. Since the velocity fields depend nonlinearly on the densities, we need to couple these errors with the density approximation error, and since the *k*-th particle is pushed forward by the approximated flow $F_{h,k}^n$ during the time interval $[t_n,t_{n+1}]$, we need to control the local error between this approximation and the exact flow $F^{t_n,t_{n+1}}$. To this end we define a first error term on the support of the smooth particles,

2.33 $$\begin{aligned} e_F^n := \sup _{k\in \mathbb Z^d} \Vert F^{t_{n},t_{n+1}} -F_{h,k}^{n} \Vert _{L^{\infty }(S^n_{h,k}) } \quad \text { with } \quad S^n_{h,k} := {{\mathrm{supp}}}\big (\varphi _{h,k}^n \big ). \end{aligned}$$

In our analysis, we shall also need to track the error on an extended domain which accounts for the deformation of the particle support by the exact flow, namely

2.34 $$\begin{aligned} \tilde{e}_F^n := \sup _{k\in \mathbb Z^d} \Vert F^{t_{n},t_{n+1}} -F_{h,k}^{n} \Vert _{L^{\infty }(\tilde{S}^n_{h,k}) } \,\, \text { with } \,\, \tilde{S}^n_{h,k} := S^n_{h,k} \cup F^{t_{n+1},t_n}(S^{n+1}_{h,k}). \end{aligned}$$

The error corresponding to the integrated flow (2.25) is then defined as

$$\begin{aligned} \overline{e_F}^n:= \sup _{k\in \mathbb Z^d} \Vert F^{0,t_{n}} -\overline{F}_{h,k}^{n} \Vert _{L^{\infty }(S^0_{h,k}) }. \end{aligned}$$

Using the fact that the exact flow is Lipschitz, see (2.6), it is easy to bound this term by accumulating the local flow errors, $\overline{e_F}^n \le C \exp (CT) (e^0_F + \cdots + e^{n-1}_F)$, but in the analysis we will need a finer control, see Lemma 4 below. We will also need to control the error of the Jacobian determinants for each particle, thus we define

2.35 $$\begin{aligned} e_j^n:= \sup _{k\in \mathbb Z^d} \left\| \frac{1}{ j^{t_{n},t_{n+1}}(x)}-\frac{1}{j_{k}^{n}} \right\| _{L^{\infty }(S^n_{h,k}) }. \end{aligned}$$

Finally we will need to track carefully the particles that affect the local value of the approximated density. For this purpose, we let

$$\begin{aligned} \mathcal {K}_{n}(x):= \{k\in \mathbb Z^d: \, x\in S^n_{h,k} \}. \end{aligned}$$

## $L^1$ and $L^\infty$ convergence for smooth potentials

In this section we assume that the potential is smooth, as defined in the introduction. This means that $\nabla W \in \mathcal {W}^{1,\infty }(\mathbb R^d)$. In this case, the Lipschitz norm of *u* is bounded by $\Vert \nabla W\Vert _{\mathcal {W}^{1,\infty }}$: indeed letting $|\cdot |$ denote the Euclidean norm in $\mathbb R^d$ as well as its associated matrix norm, we have for all $x \in \mathbb R^d$, $t \in [0,T]$,

3.1 $$\begin{aligned} \begin{aligned} |Du(t,x)|&= |(D^2 W * \rho (t)) (x)| \\&\le C \max _{1 \le i,j \le d} |(\partial _{ij} W * \rho (t))(x)| \le C \Vert \rho ^0\Vert _{L^1} \Vert \nabla W\Vert _{\mathcal {W}^{1,\infty }}. \end{aligned} \end{aligned}$$

and similarly for *u*, so that estimates (2.5)–(2.9) hold with $L = C \Vert \nabla W\Vert _{\mathcal {W}^{1,\infty }}$. However, to obtain convergence rates in $L^p$-spaces we need more regularity on the solutions. In turn we assume that $\rho ^0 \in \mathcal {W}^{1,1}_+(\mathbb R^d)$ in this section and we compute the weights with the formula (2.16) involving the dual kernels. According to the propagation of regularity of solutions to (1.1) in Proposition 9 in the “Appendix”, this ensures that the unique solution to (1.1) satisfies $\rho \in L^\infty (0,T;\mathcal {W}^{1,1}(\mathbb R^d))$ for all $T>0$.

Given the solution $\rho$ to (1.1), we will use the shortcut notation, $\rho ^n(x):= \rho (t_n,x)$ for $x \in \mathbb R^d$. From now on, *C* denotes a generic constant independent of *h* and $\Delta t$, depending only on $L= \sup _{t\le T} |u(t)|_{\mathcal {W}^{1,\infty }}$, *d* and the exact solution.

Moreover, we assume that both *h* and $\Delta t$ are bounded by an absolute constant. We denote by

3.2 $$\begin{aligned} \theta _n := \Vert \rho ^n - \rho ^n_h\Vert _{L^1}, \qquad {\tilde{\theta }}_n := \max _{0 \le m \le n} \theta _m, \quad \text{ and } \quad \varepsilon _n := \Vert \rho ^n - \rho ^n_h\Vert _{L^{\infty }} \end{aligned}$$

the errors in $L^1$ and $L^{\infty }$ norms.

In the table below, we list the most important notation used in the proofs below at both the discrete and the continuum levels together with the errors relating continuum and discrete levels.

**Table:** 

Concept	Continuum	Discrete	Error
Density	$\rho (t,x)$	$\rho ^n_h(x) = \sum _{k \in \mathbb Z^d}\omega _k \varphi ^n_{h,k}(x)$	$\theta _n$: $L^1$-error
			$\varepsilon _n$: $L^\infty$-error
			$\Gamma ^n_h$: $L^1 \cap L^p$-error
Local flow map	$F^{s,t}(x)$	$F^{n}_{h,k}(x) = x^{n+1}_k + J^n_k(x - x^n_k)$	$e^n_F$, $\tilde{e}_F^n$: $L^\infty$-errors
Iterated flow map	$F^{0,t}(x)$	$\overline{F}^n_{h,k}(x) = x^n_k + (D^n_k)^{-1}(x - x^0_k)$	$\overline{e_F}^n$: $L^\infty$-error
Jacobian matrix	$J^{s,t}(x)$	$J^n_k$	–
Deformation matrix	–	$D^{n+1}_k = D^n_k(J^n_k)^{-1}$	–
Jacobian determinant	$j^{s,t}(x)$	$j^n_k$	$e^n_j$: $L^\infty$-error
Particle volume	–	$h^{n+1}_k = j^n_k h^n_k$	–
Particle shape	–	$\varphi ^n_{h,k}(x) = \frac{1}{h^n_k}\varphi \left( \frac{D^n_k(x - x^n_k)}{h}\right)$	–

### Estimates on the flows and related terms

We first control the particle overlapping from the approximation error on the flow.

#### Lemma 1

There exists a constant *C* independent on *h* and $\Delta t$ such that

3.3 $$\begin{aligned} \kappa _n :=\sup _{x\in \mathbb R^d} \, \# \mathcal {K}_{n}(x) \le C \left( 1+ \frac{\overline{e_F}^n}{h}\right) ^d. \end{aligned}$$

#### Proof

Given $x\in \mathbb R^d$ and $k\in \mathcal {K}_{n}(x)$, we denote $z= F^{t_{n},0}(x)$ and $z_k=\big (\overline{F}_{h,k}^{n}\big )^{-1}(x)$. From (2.25) we see that $z_k \in S^0_{h,k}$. Using the Lipschitz bound (2.6) we then write

$$\begin{aligned} \begin{aligned} |z- kh |&\le |z- z_k |+ |z_k- kh | \le \left| F^{t_{n},0} \left( \overline{F}_{h,k}^{n}(z_k))- F^{0,t_{n}}(z_k) \right) \right| + |z_k-x_k^0 | \\&\le \left| F^{t_{n},0} \right| _{Lip} \overline{e_F}^n + C h \le C( \overline{e_F}^n + h). \end{aligned} \end{aligned}$$

This gives $\left| k -\frac{z}{h} \right| \le C \big ( 1 + \frac{ \overline{e_F}^n}{h}\big ),$ and the result follows. $\square$

Using the formulas (2.31), (2.32) and the a priori $L^1$ bound (2.28) on the approximated densities $\rho ^n_h$ we easily derive uniform estimates for the approximated Jacobian matrices and the particle supports.

#### Lemma 2

The approximated Jacobian determinants satisfy

$$\begin{aligned} e^{-C\Vert \Delta _x W\Vert _{L^\infty } \Delta t} \le j_k^n \le e^{C\Vert \Delta _x W\Vert _{L^\infty }\Delta t}\, , \end{aligned}$$

$J_k^n$ is always invertible and

3.4 $$\begin{aligned} e^{-C\Vert \Delta _x W\Vert _{L^\infty }T} \le \frac{h_k^n}{h^d} \le e^{C\Vert \Delta _x W\Vert _{L^\infty }T}. \end{aligned}$$

As for the deformation matrices $D^n_k = (J^{n-1}_k \cdots J^0_k)^{-1}$, they satisfy

3.5 $$\begin{aligned} \max (|D_k^n|, |(D_k^n)^{-1}|) \le e^{C\Vert D^2 W\Vert _{L^\infty }T}. \end{aligned}$$

Here, the constant *C* is uniform in *k* and $n \le N$, depending only on the $L^1$-norm of the initial data $\rho _0$.

We next show that the support of the particle approximation is of order *h*.

#### Lemma 3

If $\nabla W \in \mathcal {W}^{1,\infty }(\mathbb R^d)$, then we have

3.6 $$\begin{aligned} |x - x_k^n| \le C h \qquad \text { for } x \in S^n_{h,k} \end{aligned}$$

and

3.7 $$\begin{aligned} |x - x_k^n| \le C(h+\Delta t) \qquad \text { for } x \in \tilde{S}^n_{h,k} \end{aligned}$$

with constants *C* independent of $\Delta t$ and *h*.

#### Proof

From ${{\mathrm{supp}}}(\varphi ) \subset B(0,c)$, we easily infer that $\big |D_k^n(x-x_k^n)\big | \le c h$ holds on ${{\mathrm{supp}}}(\varphi ^n_{h,k})$, see (2.23), thus (3.6) holds for $n \le N$, using (3.5). To complete the proof we then observe that (2.1) gives

$$\begin{aligned} |x^{n+1}_k - F^{t_{n},t_{n+1}}(x^n_k)| = |\int _{t_n}^{t_{n+1}} (\nabla W * \rho _h^n)(x_k^n) - (\nabla W * \rho (\tau ))(F^{t_n,\tau }(x^n_k))d\tau | \le C \Delta t, \end{aligned}$$

so that if *x* is such that $F^{t_n,t_{n+1}}(x) \in {{\mathrm{supp}}}(\varphi ^{n+1}_{h,k})$, we have

$$\begin{aligned} \begin{aligned} |x-x^n_k|&= |F^{t_{n+1},t_n}(F^{t_n,t_{n+1}}(x)) - F^{t_{n+1},t_n}(F^{t_n,t_{n+1}}(x^n_k))| \\&\le |F^{t_n,t_{n+1}}|_{Lip} \big ( |F^{t_n,t_{n+1}}(x) - x^{n+1}_k| + |x^{n+1}_k - F^{t_{n},t_{n+1}}(x^n_k)| \big ) \\&\le C (h + \Delta t), \end{aligned} \end{aligned}$$

by using the Lipschitz estimate (2.6) and the bound (3.6) on $S^{n+1}_{h,k}$. $\square$

To control the approximation errors for the velocity and the Jacobian matrices, we next introduce the generic error

3.8 $$\begin{aligned} {\tilde{\xi }}_n(K) := \sup _{\tau \in [t_n,t_{n+1}]}\sup _{k\in \mathbb Z^d}\, \sup _{x\in \tilde{S}^n_{h,k}} \left| (K * \rho (\tau ))(F^{t_n,\tau }(x)) - (K * \rho _h^n)\ (x_k^n) \right| , \end{aligned}$$

for some given $K\in \mathcal {W}^{1,\infty }(\mathbb R^d)$ and $0\le n \le N = T / \Delta t$.

#### Proposition 4

The discrete velocity $u^n_k := -(\nabla W * \rho ^n_h)(x_k^n)$ satisfies

3.9 $$\begin{aligned} |u(\tau ,F^{t_n,\tau }(x_k^n)) - u^n_k| \le C(h^2 ||\rho ^0||_{\mathcal {W}^{1,1}} + \Delta t + \overline{e_F}^n) \end{aligned}$$

for $\tau \in [t_n,t_{n+1}]$, $0 \le n \le N-1$ and with a constant *C* independent of $\Delta t$ and *h*.

#### Proof

Using that $u(\tau ,y) = -(\nabla W * \rho (\tau ))(y) = -(\nabla W * (F^{0,\tau } \# \rho ^0))(y)$, we write

$$\begin{aligned} \begin{aligned} u(\tau ,F^{t_n,\tau }(x^n_k))&=-\int _{\mathbb R^d} \nabla W (F^{t_n,\tau }(x^n_k)-y) \rho (\tau ,y)dy \\&=-\int _{\mathbb R^d} \nabla W(F^{t_n,\tau }(x^n_k)- F^{0,\tau }(z) ) \rho ^0(z) dz \\&= (a) +(b)+(c) - \int _{\mathbb R^d} \nabla W(x_k^n-y) \rho _h^n(y)dy \end{aligned} \end{aligned}$$

with

$$\begin{aligned} \begin{aligned}&(a) := -\int _{\mathbb R^d} \left[ \nabla W(F^{t_n,\tau }(x^n_k)- F^{0,\tau }(z) ) - \nabla W(x_k^n - F^{0,t_n}(z))\right] \rho ^0(z) dz \\&(b) := -\int _{\mathbb R^d} \nabla W(x_k^n - F^{0,t_n}(z) ) \left[ \rho ^0(z) - \rho _h^0(z)\right] dz \\&(c) := -\sum _{l\in \mathbb Z^d} \omega _l \int _{S^n_{h,l}} \left[ \nabla W(x_k^n - F^{0,t_n}((\overline{F}_{h,l}^{n-1})^{-1}(y) )) - \nabla W(x_k^n-y)\right] \varphi _{h,l}^n(y)dy, \end{aligned} \end{aligned}$$

so that $\left| u(\tau ,F^{t_n,\tau }(x_k^n)) - u^n_k \right| \le |(a)|+|(b)|+|(c)|$. For the first term we write

$$\begin{aligned} |(a)| \le \Vert \nabla W\Vert _{\mathcal {W}^{1,\infty }} \int _{\mathbb R^d} |A(z)| \rho ^0(z) dz \le C ||A||_{L^\infty } \end{aligned}$$

with $A(z) := (F^{t_n,\tau }(x_k^n)-F^{0,\tau }(z)) - (x_k^n - F^{0,t_n}(z))$. Using the expression (2.1) for the exact flow, estimate (3.7) and the equality $||\rho (s)||_{L^1} = 1$ gives then

$$\begin{aligned} |A(z)| \le \int _{t_n}^\tau \left| (\nabla W*\rho (s))(F^{t_n,s}(x^n_k)) + (\nabla W*\rho (s))(F^{0,s}(z)) \right| ds \le 2 \Delta t ||\nabla W||_{L^\infty } \end{aligned}$$

so that $|(a)| \le C \Delta t$. For (*b*), using the Lipschitz regularity of the flow (2.6) and the error bound (2.19) on the initial data we find

$$\begin{aligned} \begin{aligned} |(b)|&\le e^{C T } ||\nabla W||_{\mathcal {W}^{1,\infty }} ||\rho _h^0 - \rho ^0||_{\mathcal {W}^{-1,1}} \le C h^2 ||\rho ^0||_{\mathcal {W}^{1,1}}. \end{aligned} \end{aligned}$$

Finally, we observe that for $y\in S^n_{h,l}$ we have $(\overline{F}_{h,l}^{n})^{-1}(y)\in {S^0_{h,l}}$ from (2.25), and

$$\begin{aligned} \left| F^{0,t_n}\left( (\overline{F}_{h,l}^n)^{-1}(y)\right) -y \right| \le \left| F^{0,t_n}\left( (\overline{F}_{h,l}^{n})^{-1}(y)\right) - \overline{F}_{h,l}^{n} \left( (\overline{F}_{h,l}^{n})^{-1}(y) \right) \right| \le \overline{e_F}^n, \end{aligned}$$

and arguing as in (2.28) this gives

$$\begin{aligned} \begin{aligned} |(c)|&\le \Vert \nabla W \Vert _{\mathcal {W}^{1,\infty }} \sum _{l\in \mathbb Z^d} |\omega _l| \int _{S^n_{h,l}} \left| F^{0,t_n}\left( (\overline{F}_{h,l}^{n})^{-1}(y)\right) -y \right| \varphi _{h,l}^n(y)dy \\&\le C \overline{e_F}^n \sum _{l\in \mathbb Z^d} |\omega _l| \le C \overline{e_F}^n. \end{aligned} \end{aligned}$$

By gathering the above estimates, we complete the proof. $\square$

#### Proposition 5

If the initial density satisfies $\rho ^0 \in \mathcal {W}^{1,1}_+(\mathbb R^d)$, then the estimate

$$\begin{aligned} {\tilde{\xi }}_n(D^2 W) \le C\left( \theta _n + \Delta t+h \right) \end{aligned}$$

holds with a constant *C* depending only on *d*, *T*, *L*, and $\Vert \rho ^0\Vert _{\mathcal {W}^{1,1}}$ for $\Delta t$ small enough. Moreover, at $x=x_k^n$, we have

$$\begin{aligned} \sup _{k\in \mathbb Z^d}\, \sup _{\tau \in [t_n,t_{n+1}]} \left| (D^2 W * \rho (\tau ))(F^{t_n,\tau }(x_k^n))- (D^2 W * \rho _h^n)\ (x_k^n) \right| \le C\left( \theta _n + \Delta t \right) . \end{aligned}$$

#### Proof

Given $x\in \tilde{S}^n_{h,k}$ and $\tau \in [t_n,t_{n+1}]$, we write

$$\begin{aligned} \begin{aligned}&\left| (D^2 W * \rho (\tau ))(F^{t_n,\tau }(x))- (D^2 W * \rho _h^n)\ (x_k^n) \right| \\&\qquad =\int _{\mathbb R^d} D^2 W(y)\left[ \rho (\tau ,F^{t_n,\tau }(x)-y) - \rho _h^n(x_k^n-y) \right] dy = (a)+(b), \end{aligned} \end{aligned}$$

with

$$\begin{aligned} (a):= & {} \int _{\mathbb R^d} D^2 W(y)\left[ \rho (\tau ,F^{t_n,\tau }(x)-y) - \rho (t_n,x_k^n-y) \right] dy,\\ (b):= & {} \int _{\mathbb R^d} D^2 W(y)\left[ \rho (t_n,x_k^n -y) - \rho _h^n(x_k^n-y) \right] dy. \end{aligned}$$

The second term is estimated by

$$\begin{aligned} |(b)| \le \Vert D^2 W\Vert _{L^{\infty }} \Vert \rho (t_n,\cdot )-\rho _h^n\Vert _{L^1} \le L \theta _n. \end{aligned}$$

And using $\rho (\tau ) = F^{t_n,\tau } \# \rho (t_n)$ we rewrite the first term as $(a) = (c) + (d)$ with

$$\begin{aligned} \begin{aligned} (c)&:= \int _{\mathbb R^d} D^2 W(y)\rho (t_n,F^{\tau ,t_n}(F^{t_n,\tau }(x)-y))\left[ j^{\tau ,t_n}(F^{t_n,\tau }(x)-y) -1 \right] dy\\(d)&:= \int _{\mathbb R^d} D^2 W(y)[\rho (t_n,F^{\tau ,t_n}(F^{t_n,\tau }(x)-y))- \rho (t_n,x_k^n-y)]\,dy. \end{aligned} \end{aligned}$$

For (*c*) we use the one-to-one mapping $\Phi : y \mapsto F^{\tau ,t_n}(F^{t_n,\tau }(x)-y)$ with Jacobian determinant $|\det \Phi (y)| = j^{\tau ,t_n}(F^{t_n,\tau }(x)-y)$. The change of variable formula yields

$$\begin{aligned} \int _{\mathbb R^d} \rho (t_n,F^{\tau ,t_n}(F^{t_n,\tau }(x)-y)) dy \le C \int _{\mathbb R^d} \rho (t_n,\Phi (y)) |\det \Phi (y)| dy = C ||\rho (t_n)||_{L^1} \le C \end{aligned}$$

where we have used (2.8) in the first inequality. Using (2.9) this allows to bound

$$\begin{aligned} |(c)| \le C\Delta t \Vert D^2 W\Vert _{L^\infty } \int _{\mathbb R^d} \rho (t_n,F^{\tau ,t_n}(F^{t_n,\tau }(x)-y)) dy \le C \Delta t. \end{aligned}$$

Turning next to the (*d*) term, we introduce

$$\begin{aligned} \Xi _\alpha : y \mapsto \alpha (F^{\tau ,t_n}(F^{t_n,\tau }(x)-y)) + (1-\alpha )(x_k^n-y) \quad \text {for} \quad \alpha \in [0,1], \end{aligned}$$

so that

$$\begin{aligned} \begin{aligned} |(d)|&\le \Vert D^2 W\Vert _{L^\infty }\int _{\mathbb R^d} |\rho (t_n,\Xi _1(y)) - \rho (t_n,\Xi _0(y))| dy \\&\le C \int _{\mathbb R^d} \int _0^1 |\nabla \rho (\Xi _\alpha (y))| |F^{\tau ,t_n} (F^{t_n,\tau }(x)-y) -(x_k^n-y)| \, d\alpha dy \\&\le C (h + \Delta t) \int _{\mathbb R^d} \int _0^1 |\nabla \rho (\Xi _\alpha (y))| \, d\alpha dy \end{aligned} \end{aligned}$$

where in the last inequality we have used (see (2.1) and Lemma 3)

$$\begin{aligned} \begin{aligned}&|F^{\tau ,t_n} (F^{t_n,\tau }(x)-y) - (x_k^n-y)| \\&\quad = \left| F^{t_n,\tau }(x)-x^n_k -\int _{\tau }^{t_n} (\nabla W * \rho (s))(F^{\tau ,s}(F^{t_n,\tau }(x)-y))ds \right| \\&\quad \le (|x-x^n_k| + 2\Delta t \Vert \nabla W\Vert _{L^\infty }) \le C (h+ \Delta t). \end{aligned} \end{aligned}$$

To end the proof we will show that up to a sign and a translation, $\Xi _\alpha$ is uniformly close to the identity mapping. Let $G(y) := (F^{\tau ,t_n} - I)(F^{t_n,\tau }(x)-y)$ so that $\Xi _\alpha (y) = -y + \alpha G(y) + (1-\alpha ) x^n_k + \alpha F^{t_n,\tau }(x)$. From (2.7) we infer

$$\begin{aligned} |DG(y)| = |I_d - J^{\tau ,t_n}(F^{t_n,\tau }(x)-y)| \le C \Delta t \end{aligned}$$

hence there exists a constant $\gamma$ independent of *h*, $\Delta t$ and *n*, such that

$$\begin{aligned} |G(y)-G(y')| \le \gamma \Delta t |y-y'|. \end{aligned}$$

This shows that $\Xi _\alpha$ is injective for $\Delta t$ small enough, indeed if $\Xi _\alpha (y) = \Xi _\alpha (y')$ for $y \ne y'$ then $y-y' = \alpha (G(y)-G(y'))$ leads to a contradiction for $\gamma \Delta t < 1$. Moreover, using $D \Xi _\alpha (y) = -I_d + \alpha DG(y)$ and the Jacobi formula for $\partial _\alpha \det (D \Xi _\alpha )$ we find

$$\begin{aligned} |\det (D \Xi _\alpha )(y) + 1| \le C \Delta t, \end{aligned}$$

which shows that for $\Delta t$ small enough, $|\det (D \Xi _\alpha )|$ is bounded from below by a positive constant $\tilde{\gamma }$. Using again the change of variable theorem this gives

$$\begin{aligned} {\tilde{\gamma }} \int _{\mathbb R^d} |\nabla \rho (\Xi _\alpha (y))| dy \le \int _{\mathbb R^d} |\nabla \rho (\Xi _\alpha (y))| |\det (D \Xi _\alpha )(y)| dy \le \int _{\mathbb R^d} |\nabla \rho (z)| dz \le ||\rho ||_{\mathcal {W}^{1,1}}. \end{aligned}$$

The desired bound $|d| \le C(h+\Delta t)$ follows by gathering the above steps. $\square$

We can now compute an estimate for the error of the Jacobian determinants.

#### Corollary 1

Assume that $\rho ^0 \in \mathcal {W}^{1,1}_+(\mathbb R^d)$, then the following estimate holds

3.10 $$\begin{aligned} e_j^n \le C \Delta t\left( \theta _n+\Delta t+ h \right) \quad \text{ for } \text{ all } \quad 0 \le n \le N, \end{aligned}$$

for $\Delta t$ small enough, where *C* is a positive constant depending only on *T*, *L*, and $\Vert \rho \Vert _{L^\infty (0,T:\mathcal {W}^{1,1})}$.

#### Proof

According to (2.4) and (2.32), we have

$$\begin{aligned} \begin{aligned} \frac{1}{j_k^n} - \frac{1}{j^{t_n,t_{n+1}}(x)}&= \exp (\beta ^n_k) - \exp (\beta ^n(x)) \\&= (\beta ^n_k-\beta ^n(x)) \int _0^1 \exp \big (r\beta ^n_k + (1-r) \beta ^n(x)\big ) dr \end{aligned} \end{aligned}$$

with $\beta ^n_k := \Delta t (\Delta _x W * \rho _h^n)(x_k^n)$ and $\beta ^n(x) := \int _{t_n}^{t_{n+1}} (\Delta _x W * \rho (\tau ))(F^{t_n,\tau }(x)) d\tau$. Since $e_j^n$ involves the above difference for $x\in S^n_{h,k} \subset \tilde{S}^n_{h,k}$, see (2.35), we infer from (3.8) that $|\beta ^n_k - \beta ^n(x)| \le C \Delta t \, {\tilde{\xi }}_n(D^2 W)$. Using the $L^1$ bound (2.28) on $\rho ^n_h$ this yields

$$\begin{aligned} e_j^n \le C \Delta t \, {\tilde{\xi }}_n(D^2 W) \exp \left( C \Delta t \Vert \Delta _x W\Vert _{L^\infty } \right) , \end{aligned}$$

so that Proposition 5 gives the desired result. $\square$

From Proposition 5 we also derive an estimate for the error between Jacobian matrices.

#### Corollary 2

If $\rho ^0 \in \mathcal {W}^{1,1}_+(\mathbb R^d)$, then for $0 \le n \le N$ the following estimate holds

$$\begin{aligned} |J_k^n - J^{t_n,t_{n+1}}(x)| \le C\Delta t\left(\theta _n + h + \Delta t\right) \qquad \text{ for } x \in S^n_{h,k}, \end{aligned}$$

for $\Delta t$ small enough, with a constant *C* independent of $\Delta t$ and *h*. At $x = x_k^n$, we have

3.11 $$\begin{aligned} |J_k^n - J^{t_n,t_{n+1}}(x_k^n)| \le C\Delta t\left(\theta _n + \Delta t\right). \end{aligned}$$

#### Proof

Using the matrix $\tilde{J}^{t_n,t_{n+1}}(x)$ defined by (2.29), Proposition 3 gives

$$\begin{aligned} |J_k^n - J^{t_n,t_{n+1}}(x)| \le |J_k^n - \tilde{J}^{t_n,t_{n+1}}(x)| + C (\Delta t)^2 \end{aligned}$$

and to bound the remaining error we proceed as in the proof of Corollary 1: denoting $B^n_k := -\Delta t \,(D^2W * \rho _h^n)(x_k^n)$ and $B^n(x) := -\int _{t_n}^{t_{n+1}} (D^2 W * \rho (\tau ))(F^{t_n,\tau }(x)) d\tau$, we use the exponential matrix expressions (2.31) and (2.29) to compute

$$\begin{aligned} \begin{aligned} J_k^n - J^{t_n,t_{n+1}}(x)&= \exp (B^n_k) - \exp (B^n(x)) \\&= (B^n_k-B^n(x)) \int _0^1 \exp \big (rB^n_k + (1-r) B^n(x)\big ) dr. \end{aligned} \end{aligned}$$

For $x\in S^n_{h,k}$ we have $|B^n_k - B^n(x)| \le C \Delta t \, \xi _n(D^2 W)$ and using (2.28) this yields

$$\begin{aligned} |J_k^n - J^{t_n,t_{n+1}}(x)| \le C \Delta t \, \xi _n(D^2 W) \exp \left( C \Delta t \Vert D^2 W\Vert _{L^\infty } \right) \end{aligned}$$

so that the desired result follows again from Proposition 5. $\square$

#### Remark 2

If $\rho ^0$ is only assumed to be an $L^1(\mathbb R^d)$ function (or a Radon measure), then $\xi _n(D^2 W)$ can be bounded by a constant using the $L^1$ bound on $\rho ^n_h$, see (2.28), and the $\mathcal {W}^{1,\infty }(\mathbb R^d)$ smoothness of $\nabla W$. Arguing as in the proof above we then find an error estimate for the Jacobian matrices on the order of $\Delta t$.

We next turn to the approximation errors involving the forward characteristic flows and we establish a series of estimates.

#### Lemma 4

For $0 \le n\le N-1$, the following estimate holds

3.12 $$\begin{aligned} \overline{e_F}^{n+1} \le e^{C\Delta t}\overline{e_F}^n + \tilde{e}_F^n \end{aligned}$$

with a constant *C* independent of $\Delta t$ and *h*.

#### Proof

Given $x\in S^0_{k,h}$ we write $y=F^{0,t_n}(x)$ and $\tilde{y}_k= \overline{F}_{h,k}^n(x) \in S^n_{h,k}$. We have

$$\begin{aligned} \begin{aligned} \left| \overline{F}_{h,k}^{n+1}(x) - F^{0,t_{n+1}}(x) \right|&= \left| F_{h,k}^n(\tilde{y}_k) - F^{t_n,t_{n+1}}(y)\right| \\&\le \left| F^{t_n,t_{n+1}}( \tilde{y}_k )- F^{t_n,t_{n+1}}(y)\right| + \left| F^{t_n,t_{n+1}}( \tilde{y}_k ) - F_{h,k}^n(\tilde{y}_k)\right| \\&\le \left| F^{t_n,t_{n+1}} \right| _{Lip}| \tilde{y}_k -y| + \Vert F^{t_n,t_{n+1}} - F_{h,k}^n\Vert _{L^{\infty }(S^n_{h,k})} \\&\le e^{C\Delta t} \overline{e_F}^n+ \tilde{e}_F^n \end{aligned} \end{aligned}$$

by using $S^n_{h,k} \subset \tilde{S}^n_{h,k}$ and the Lipschitz bound (2.6) on the exact flow. $\square$

#### Proposition 6

If $\rho ^0 \in \mathcal {W}^{1,1}_+(\mathbb R^d)$, then the following estimate holds

3.13 $$\begin{aligned} \tilde{e}_F^{n } \le C \Delta t( \Delta t + h^2+ (h+\Delta t) \theta _n + \overline{e_F}^n) \quad \text{ for } \quad 0 \le n \le N, \end{aligned}$$

for $\Delta t$ small enough, with a constant *C* independent of $\Delta t$ and *h*.

#### Proof

Given $x\in \tilde{S}^n_{h,k}$, we rewrite the linearized flow (2.22) as follows,

$$\begin{aligned} \begin{aligned} F_{h,k}^n(x)&= F_{h,k}^n(x_k^n) + J_{k}^n(x -x_k^n) = (a) + (b) + (c) + F^{t_n,t_{n+1}}(x) \end{aligned} \end{aligned}$$

with

$$\begin{aligned} \begin{aligned}&(a) := F_{h,k}^n(x_k^n) - F^{t_n,t_{n+1}}(x_k^n) \\&(b) := \left( J_{k}^n- J^{t_n,t_{n+1}}(x_k^n) \right) (x -x_k^n) \\&(c) := F^{t_n,t_{n+1}}(x_k^n) + J^{t_n,t_{n+1}}(x_k^n)(x-x_k^n)-F^{t_n,t_{n+1}}(x). \end{aligned} \end{aligned}$$

Using (2.31) and the expression (2.1) for the exact flow, we then compute

$$\begin{aligned} |(a)| = \int _{t_n}^{t_{n+1}} \left| (\nabla W * \rho ^n_h)(x_k^n) + u(\tau , F^{t_n,\tau }(x_k^n)) \right| d\tau \le C\Delta t \left( h^2 + \Delta t + \overline{e_F}^n \right) \end{aligned}$$

where the inequality follows from (3.9) (note that here *C* depends on $||\rho ^0||_{\mathcal {W}^{1,1}}$). For (*b*), we easily get using estimate (3.11) in Corollary 2 and Lemma 3 that

$$\begin{aligned} \begin{aligned} |(b)| \le |J_k^n - J^{t_n,t_{n+1}}(x_k^n)||x -x_k^n| \le C \Delta t (\theta _n+\Delta t )(h + \Delta t). \end{aligned} \end{aligned}$$

Turning to (*c*) we next differentiate (2.3) and obtain for $1 \le i,j,m \le d$,

$$\begin{aligned}\begin{aligned} \partial _m\left(J^{t_n,t_{n+1}}\right)_{ij}&= -\sum _{l = 1}^d\int _{t_n}^{t_{n+1}} (\partial _{il} W * \nabla \rho (\tau ))(F^{t_n,\tau }(x))\partial _m F^{t_n,\tau }(x)\left(J^{t_n,\tau }(x)\right)_{lj} d\tau \\&\quad -\sum _{l=1}^d \int _{t_n}^{t_{n+1}} (\partial _{il}W * \rho (\tau ))(F^{t_n,\tau }(x))\partial _m\left(J^{t_n,\tau }(x)\right)_{lj} d\tau . \end{aligned}\end{aligned}$$

This yields

$$\begin{aligned} \begin{aligned} |\partial _m J^{t_n,t_{n+1}}(x)| \le C\Delta t + C\int _{t_n}^{t_{n+1}} |\partial _m J^{t_n,\tau }(x)| d\tau , \end{aligned}\end{aligned}$$

where we used that $\rho \in L^\infty (0,T;\mathcal {W}^{1,1}(\mathbb R^d))$, $\nabla W \in \mathcal {W}^{1,\infty }(\mathbb R^d)$ and $|\partial _m F^{t_n,\tau }| \le C$ for some *C*, see (2.5). Invoking the Gronwall Lemma, we then obtain

$$\begin{aligned} |\partial _m J^{t_n,t_{n+1}}(x)| \le C\Delta t e^{C\Delta t}, \qquad m = 1, \cdots , d, \end{aligned}$$

where *C* only depends on *d*, *T*, *L* and $||\rho ^0||_{\mathcal {W}^{1,1}}$. With a Taylor expansion this gives

$$\begin{aligned} |(c)|\le \frac{1}{2} \left| D^2 F^{t_n,t_{n+1}} (\eta _k^n)\right| |x-x_k^n|^2 \le C \Delta t (h+\Delta t)^2 \end{aligned}$$

for some $\eta _k^n$ between *x* and $x_{k}^n$ and a constant *C* that only depends on *d*, *T*, *L* and $||\rho ^0||_{\mathcal {W}^{1,1}}$. Combining the above estimates yields the desired result. $\square$

We finally provide estimates for $\overline{e_F}^n$ and $\tilde{e}_F^n$.

#### Corollary 3

If $\rho ^0 \in \mathcal {W}^{1,1}_+(\mathbb R^d)$, then the following estimates hold for $0 \le n \le N$,

$$\begin{aligned} \overline{e_F}^{n} \le C(h^2 + \Delta t + h{\tilde{\theta }}_{n-1}) \quad \text {and} \quad \tilde{e}_F^n \le C\Delta t (h^2 + \Delta t + h{\tilde{\theta }}_{n-1}), \end{aligned}$$

for $\Delta t$ small enough, with ${\tilde{\theta }}_n := \max _{m\le n} \theta _m$, see (3.2), and a constant *C* independent of $\Delta t$ and *h*.

#### Proof

Using (3.12), (3.13) and the fact that $e^{C\Delta t} + C\Delta t \le e^{2C\Delta t}$, we find

$$\begin{aligned} \overline{e_F}^{n+1} \le e^{2C\Delta t}\overline{e_F}^n + C\Delta t(h^2 + \Delta t + h{\tilde{\theta }}_n), \end{aligned}$$

hence

$$\begin{aligned} \overline{e_F}^{n+1} \le e^{2CN\Delta t} (\overline{e_F}^0 + N \Delta t (h^2 + \Delta t + h{\tilde{\theta }}_n)) \le C(h^2 + \Delta t + h{\tilde{\theta }}_n), \quad n\le N-1, \end{aligned}$$

follows by a summation using $\overline{e_F}^0 = 0$. The bound on $\tilde{e}_F^n$ is obtained with (3.13). $\square$

### Proof of $L^1$ and $L^{\infty }$ convergence results

#### Theorem 1

Assume $\Delta t = O(h)$ and $\Delta t$ small enough. If $\rho ^0 \in \mathcal {W}^{1,1}_+(\mathbb R^d)$ and $\nabla W \in \mathcal {W}^{1,\infty }(\mathbb R^d)$, then

$$\begin{aligned} \max _{0\le n\le N}\Vert \rho (t_n) - \rho ^n_h\Vert _{L^1} \le C \left( \Vert \rho ^0 - \rho ^0_h\Vert _{L^1} +\frac{\Delta t}{h} + h \right) \end{aligned}$$

holds with a constant *C* depending only on *d*, *T*, *L*, and $||\rho ^0||_{\mathcal {W}^{1,1}}$.

#### Remark 3

From this result, it is clear that we need a restrictive constraint on $\Delta t=o(h)$ for convergence. This is a consequence of the low order time discretization considered in Sect. 2.3, implying the need of small time stepping. As for the factor $\frac{1}{h}$, it comes from the Lipschitz constant of the particle shape functions, and it is classical in the analysis of particle methods. It is not clear how to improve these error estimates even if high order time integrators are used to improve the ODE solver for the particle positions.

#### Proof

Let $y\in \mathbb R^d$. Using the relation $\rho (t_n) = F^{t_n,t_{n-1}} \# \rho (t_{n-1})$ and the form (2.27) of the approximate solution together with the fact that $h^n_k = h^{n-1}_k j^{n-1}_k$, we decompose the error $\rho (t_n,y) - \rho _h^n(y)$ into three parts as

3.14 $$\begin{aligned} \begin{aligned} \rho (t_n,y) - \rho _h^n(y)&= \underbrace{ \left[ \rho \left( t_{n-1}, F^{t_{n},t_{n-1}}(y)\right) - \rho _h^{n-1}\left( F^{t_n,t_{n-1}}(y)\right) \right] j^{t_n,t_{n-1}}(y) }_{A_n(y)} \\&\quad + \underbrace{ \sum _{k\in \mathbb Z^d} \frac{\omega _k }{h_k^{n-1}} \varphi \left( \frac{D_k^{n-1}}{h}\left( F^{t_n,t_{n-1}}(y)- x_k^{n-1}\right) \right) \left[ j^{t_n,t_{n-1}}(y) - \frac{1}{j_{k}^{n-1}} \right] }_{B_n(y)} \\&\quad + \underbrace{\sum _{k\in \mathbb Z^d} \frac{\omega _k }{h_k^n} \left[ \varphi \left( \frac{D_k^{n-1}}{h} (F^{t_n,t_{n-1}}(y)- x_k^{n-1} ) \right) - \varphi \left( \frac{D_k^{n}}{h} (y- x_k^{n}) \right) \right] }_{C_n(y)}. \end{aligned} \end{aligned}$$

$\diamond$ Estimate of $\Vert A_n\Vert _{L^1}$: Using the one-to-one change of variable $x= F^{t_{n},t_{n-1}}(y)$, we easily find that

$$\begin{aligned} \int _{\mathbb R^d} |A_n(y)|dy = \int _{\mathbb R^d} | \rho (t_{n-1},x)-\rho _h^{n-1}(x)|dx=\theta _{n-1}. \end{aligned}$$

$\diamond$ Estimate of $\Vert B_n\Vert _{L^1}$: By means of the same change of variable and the relation $j^{t_n,t_{n-1}}(y) = (j^{ t_{n-1},t_n}(x))^{-1}$, we obtain

$$\begin{aligned} \begin{aligned} \int _{\mathbb R^d} |B_n(y)|dy&\le \int _{\mathbb R^d} \sum _{k\in \mathbb Z^d} |\omega _k| \varphi _{h,k}^{n-1}(x) \left| \frac{1}{ j^{ t_{n-1},t_n}(x) } - \frac{1}{j_{k}^{n-1}} \right| j^{t_{n-1},t_n}(x)dx\\&\le e_{j}^{n-1} || j^{ t_{n-1},t_n}||_{L^\infty } \int _{\mathbb R^d}\sum _{k\in \mathbb Z^d} | \omega _k| \varphi _{h,k}^{n-1}(x) dx \le C e_{j}^{n-1}, \end{aligned} \end{aligned}$$

due to (2.5), (2.28) and (2.35), indeed *x* can be taken in $S^{n-1}_{h,k}$ in the *k*-th term.

$\diamond$ Estimate of $\Vert C_n\Vert _{L^1}$: Writing again $x = F^{t_{n},t_{n-1}}(y)$, we observe that in the *k*-th term, we must consider the cases where $y \in S^n_{h,k}$ and those where $x \in S^{n-1}_{h,k}$. Thus, *x* must be taken in the extended particle support $\tilde{S}^{n-1}_{h,k}$, see (2.34). Using the incremental relation (2.24) we then estimate

$$\begin{aligned} |D_k^{n-1} (x - x_k^{n-1} ) - D_k^{n} (y- x_k^{n})|= & {} |D^n_k (x_k^{n} + J^{n-1}_k(x-x^{n-1}_k) - F^{t_{n-1},t_n}(x))|\\\le & {} |D^n_k| \tilde{e}^{n-1}_F \end{aligned}$$

see (2.22), (2.33). To obtain a global bound we next observe that the measure of $\tilde{S}^{n-1}_{h,k}$ is of order $(h+\Delta t)^d \le Ch^d$ according to Lemma 3 and the assumption $\Delta t \le C h$, as well as that of $F^{t_{n-1},t_{n}}(\tilde{S}^{n-1}_{h,k})$ according to (2.8). Using the above observations and the fact that the reference shape $\varphi$ is assumed to be Lipschitz, we find

3.15 $$\begin{aligned} \int _{\mathbb R^d} |C_n(y)|dy \le C h^d \sum _{k \in \mathbb Z^d} \frac{|\omega _k|}{h_k^n} \frac{|D^n_k|}{h} \tilde{e}^{n-1}_F \le C \frac{\tilde{e}^{n-1}_F}{h}, \end{aligned}$$

where the last inequality follows from the uniform bounds on the matrices $D^n_k$ and their determinants (Lemma 2), and from the estimates inside (2.28).

$\diamond$ Conclusion: We now combine all the estimates above and (3.10) in Corollary 1 to obtain

$$\begin{aligned} \theta _n \le \theta _{n-1} + Ce_j^{n-1} + C\frac{\tilde{e}_F^{n-1}}{h} \le (1 + C\Delta t)\theta _{n-1} + C\Delta t(\Delta t + h) + C\frac{\tilde{e}_F^{n-1}}{h}. \end{aligned}$$

Using Corollary 3 to estimate the flow error yields

$$\begin{aligned} {\tilde{\theta }}_n \le (1 + C\Delta t){\tilde{\theta }}_{n-1} + C\Delta t \left( \Delta t + h +\frac{\Delta t}{h}\right) . \end{aligned}$$

Since $h\le 1$, we conclude that

$$\begin{aligned} {\tilde{\theta }}_n \le e^{CN\Delta t}\theta _0 + e^{CN\Delta t}\left( h+\frac{\Delta t}{h}\right) \le C\left( h+\theta _0 + \frac{\Delta t}{h}\right) . \end{aligned}$$

$$\square $$

We next derive $L^\infty$-estimates. Here the required regularity propagates in time. As proved in the “Appendix”, Proposition 9, the unique solution to (1.1) belongs to $\rho \in L^\infty (0,T;(\mathcal {W}^{1,1}_+\cap L^\infty )(\mathbb R^d))$ provided that $\rho ^0 \in (\mathcal {W}^{1,1}_+ \cap L^\infty )(\mathbb R^d)$.

#### Theorem 2

If $\Delta t = O(h)$ and $\Delta t$ small enough, $\rho ^0 \in \mathcal {W}^{1,1}_+(\mathbb R^d)\cap L^\infty (\mathbb R^d)$ and $\nabla W \in \mathcal {W}^{1,\infty }(\mathbb R^d)$, then

$$\begin{aligned} \max _{0\le n\le N}\Vert \rho (t_n) - \rho ^n_h\Vert _{L^{\infty }} \le C\left( h + \Vert \rho ^0 - \rho ^0_h\Vert _{L^{\infty }}+ \Vert \rho ^0 - \rho ^0_h\Vert _{L^{1}} + \frac{\Delta t}{h}\right) \end{aligned}$$

holds with a constant independent of *h* and $\Delta t$.

#### Proof

Given $y\in \mathbb R^d$, we decompose $\rho (t_n,y) - \rho _h^n(y)$ into three terms as in (3.14).

$\diamond$ Estimate of $\Vert A_n\Vert _{L^\infty }$: Using the bound (2.8) on the exact Jacobian determinant, we find

$$\begin{aligned} \Vert A_n\Vert _{L^{\infty }} \le e^{C\Delta t} \varepsilon _{n-1}. \end{aligned}$$

$\diamond$ Estimate of $\Vert B_n\Vert _{L^\infty }$: Writing again $x = F^{t_{n},t_{n-1}}(y)$, we observe that the *k*-th term vanishes if $x \not \in S^{n-1}_{h,k}$. In particular, the sum can be restricted to the indices *k* in the set $\mathcal {K}_{n-1}(x)$. Gathering the bounds (3.4) on $h^n_k$, (2.20) on $\omega _k$ and (3.3) on $\kappa _n := \sup _{x\in \mathbb R^d} \#(\mathcal {K}_{n-1}(x))$, we compute

$$\begin{aligned} |B_n(y)| \le C \#(\mathcal {K}_{n-1}(x)) ||\rho ^0||_{L^\infty } ||\varphi ||_{L^\infty } e^{n-1}_j \le C \left( 1+ \frac{\overline{e_F}^n}{h}\right) ^d e^{n-1}_j. \end{aligned}$$

$\diamond$ Estimate of $\Vert C_n\Vert _{L^\infty }$: Similarly as in the proof of Theorem 1, we observe that the *k*-th summand in $C_n(y)$ must be considered when $y \in S^n_{h,k}$ or when $x \in S^{n-1}_{h,k}$ (or both). Clearly the cardinality of the corresponding index set satisfies

$$\begin{aligned} \#(\{k \in \mathbb Z^d : y \in S^n_{h,k} \text { or } x \in S^{n-1}_{h,k}\}) \le \#(\mathcal {K}_n(y)) + \#(\mathcal {K}_{n-1}(x)) \le \kappa _n + \kappa _{n-1}. \end{aligned}$$

Using the Lipschitz smoothness of the reference shape function $\varphi$ as in (3.15), and again the bounds (3.4) on $h^n_k$, (2.20) on $\omega _k$ and (3.3) on $\kappa _n$, we write

$$\begin{aligned} |C_n(y)| \le C ( \kappa _n + \kappa _{n-1}) \frac{\tilde{e}^{n-1}_F}{h} \le C \left( \left(1+ \frac{\overline{e_F}^n}{h}\right)^d + \left(1+\frac{\overline{e_F}^{n-1}}{h}\right)^d\right) \frac{\tilde{e}_F^{n-1}}{h}. \end{aligned}$$

$\diamond$ Conclusion: Combining the estimates above, we have

3.16 $$\begin{aligned} \varepsilon _n \le e^{C\Delta t} \varepsilon _{n-1}+ C\left( 1+\frac{\overline{e_F}^{n-1} }{h} \right) ^d e_{j}^{n-1} + C\left(1+ \frac{\overline{e_F}^n + \overline{e_F}^{n-1}}{h} \right)^d \frac{\tilde{e}_F^{n-1}}{h}. \end{aligned}$$

Now, with the assumptions made here Theorem 1 applies, hence Corollaries 1 and 3 provide error estimates for the Jacobian and flow errors. Specifically, we have

$$\begin{aligned} e_j^{n-1} \le C\Delta t(\Delta t + h ), \quad \overline{e_F}^n \le C(h^2 +\Delta t+h\theta _0), \quad \tilde{e}_F^{n-1} \le C\Delta t(h^2 +\Delta t+h\theta _0). \end{aligned}$$

Plugging these estimates into (3.16) yields then

$$\begin{aligned} \varepsilon _n \le e^{C \Delta t} \varepsilon _{n-1} + C\Delta t\left( h+\theta _0 +\frac{\Delta t}{h}\right) , \end{aligned}$$

due to $\Delta t \lesssim h \lesssim 1$ and $\theta _0 \le 2$. We again conclude with the discrete Gronwall Lemma. $\square$

## Convergence for measure solutions with smooth potentials

In this part, we consider measure valued solutions to the system (1.1) using the bounded Lipschitz distance. More precisely, let $\rho _1,\rho _2 \in \mathcal {M}(\mathbb R^d)$ be two Radon measures. Then the bounded Lipschitz distance $d_{BL}(\rho _1,\rho _2)$ between $\rho _1$ and $\rho _2$ is given by

$$\begin{aligned} d_{BL}(\rho _1,\rho _2) : = \sup \left\{ \left| \int _{\mathbb R^d} \psi d\rho _1 -\int _{\mathbb R^d}\psi d\rho _2 \right| : \psi \in \mathcal {W}^{1,\infty }(\mathbb R^d) ~~ \text{ and } ~~ \Vert \psi \Vert _{\mathcal {W}^{1,\infty } }\le 1 \right\} . \end{aligned}$$

Since the interaction potential *W* satisfies $\nabla W \in \mathcal {W}^{1,\infty }(\mathbb R^d)$, a well-posedness theory for measure valued solutions to (1.1) can be developed by using the classical results of Dobrushin [45], see [24, 53] for related results.

To estimate the error between the exact flow and its local linearizations we now revisit some results from the previous section, namely Proposition 6, given the low regularity of the solutions. As in the previous section, we denote $\rho ^n = \rho (t_n)$.

### Proposition 7

Let $\rho ^0$ be an initial Radon measure on $\mathbb R^d$, and $\rho ^n_h$ be the approximation constructed in (2.27). If *W* satisfies $\nabla W \in \mathcal {W}^{1,\infty }(\mathbb R^d)$, then the flow error defined on the particles support (2.33)

$$\begin{aligned} e_F^n \le C\Delta t\left(d_{BL}(\rho ^n, \rho ^n_h) + h + \Delta t \right) \end{aligned}$$

holds for $0 \le n \le N$ with a constant *C* independent of *h* and $\Delta t$.

### Proof

Let $x\in S^n_{h,k}$. We decompose the linearized flow as in Proposition 6,

$$\begin{aligned} F_{h,k}^n(x) = F_{h,k}^n(x_k^n) + J_{k}^n(x -x_k^n) = (a) + (b) + (c) + F^{t_n,t_{n+1}}(x) \end{aligned}$$

with

$$\begin{aligned} \begin{aligned}&(a) := F_{h,k}^n(x_k^n) - F^{t_n,t_{n+1}}(x_k^n) \\&(b) := \left( J_{k}^n- J^{t_n,t_{n+1}}(x_k^n) \right) (x -x_k^n) \\&(c) := F^{t_n,t_{n+1}}(x_k^n) + J^{t_n,t_{n+1}}(x_k^n)(x-x_k^n)-F^{t_n,t_{n+1}}(x). \end{aligned} \end{aligned}$$

We next rewrite $(a) = \int _{t_n}^{t_{n+1}} \left( (\nabla W * \rho ^n_h)(x_k^n) - (\nabla W * \rho (\tau ))(F^{t_n,\tau }(x_k^n))\right)d\tau$ using (2.31) and (2.1), and estimate the integrand by

$$\begin{aligned} \begin{aligned}&(\nabla W * \rho ^n_h)(x_k^n) - (\nabla W * \rho (\tau ))(F^{t_n,\tau }(x_k^n))\\&\qquad = \int _{\mathbb R^d} \nabla W(x_k^n - y)\rho ^n_h(y) - \nabla W(F^{t_n,\tau }(x_k^n) - y)\rho (\tau ,y)\,dy \\&\qquad = \int _{\mathbb R^d} \nabla W(x_k^n - y)\left( \rho ^n_h(y) - \rho ^n(y)\right)dy \\&\qquad \quad + \int _{\mathbb R^d} \nabla W(x_k^n - y)\rho ^n(y) - \nabla W(F^{t_n,\tau }(x_k^n) - y)\rho (\tau ,y)\,dy \\&\qquad =: (d) + (e). \end{aligned}\end{aligned}$$

From $\nabla W \in \mathcal {W}^{1,\infty }(\mathbb R^d)$, we infer $|(d)| \le C d_{BL}(\rho ^n, \rho ^n_h)$. Using next a change of variable and the relation $\rho (\tau ) = F^{t_{n},\tau } \# \rho ^{n}$ we get

$$\begin{aligned}\begin{aligned} (e)&= \int _{\mathbb R^d} \left(\nabla W(x_k^n - y) - \nabla W(F^{t_n,\tau }(x_k^n) - F^{t_n,\tau }(y))\rho ^n(y)\right)dy\\&\le \int _{\mathbb R^d} \Vert D^2 W\Vert _{L^\infty }\left|x_k^n - y - (F^{t_n,\tau }(x_k^n) - F^{t_n,\tau }(y)) \right|\rho ^n(y)\,dy \le C\Delta t. \end{aligned}\end{aligned}$$

Combining the estimates above, we obtain

$$\begin{aligned} |(a)| \le C\Delta t\left(d_{BL}(\rho ^n, \rho ^n_h) + \Delta t \right). \end{aligned}$$

For the estimate of (*b*), we easily get from Remark 2 that $|(b)| \le C h \Delta t$. Finally, we observe that (*c*) cannot be estimated as in the proof of Proposition 6, due to the lesser regularity of the densities. We then proceed as follows,

$$\begin{aligned}\begin{aligned} |(c)|&= \left| (x_k^n -x)\left( I_{d}- J^{t_n,t_{n+1}}(x_k^n)\right) + \int _{t_n}^{t_{n+1}}\left[ u(\tau , F^{t_n,\tau }( x_k^n))- u(\tau , F^{t_n,\tau }( x )) \right] d\tau \right| \\&\le |x_k^n-x| \Vert I_{d} - J^{t_n,t_{n+1}}(\cdot ) \Vert _{L^{\infty }} + \Vert D^2 W\Vert _{L^\infty } \int _{t_n}^{t_{n+1}} \left| F^{t_n,\tau }( x_k^n) -F^{t_n,\tau }( x ) \right| d\tau \\&\le C h \, \Delta t + C \int _{t_n}^{t_{n+1}} \left| F^{t_n,\tau } \right| _{Lip} |x_k^n-x | d\tau \le C h \, \Delta t, \end{aligned}\end{aligned}$$

where we used estimate (3.6) for $x \in S^n_{h,k}$, and the estimates (2.6) and (2.7). $\square$

We now show that our LTP method is unconditionally stable in the weak norm between measures $d_{BL}$.

### Theorem 3

Let $\rho ^0$ be an initial probability measure on $\mathbb R^d$, and $\rho ^n_h$ be the approximation constructed in (2.27). Assume that the interaction potential *W* satisfies $\nabla W \in \mathcal {W}^{1,\infty }(\mathbb R^d)$, then the estimate

$$\begin{aligned} \max _{0\le n\le \tfrac{T}{\Delta t}} d_{BL}(\rho ^n,\rho ^n_h) \le C(d_{BL}(\rho ^0,\rho ^0_h) + h + \Delta t) \end{aligned}$$

holds, where *C* depends only on *d*, *L* and *T*.

### Remark 4

Observe that a convergence condition on the approximation of the initial data in Theorem 3 such as $d_{BL}(\rho ^0,\rho ^0_h) \lesssim h$ is easily achieved by using a uniform quadrangular mesh of size $h^d$ and approximating the initial data $\rho ^0$ by a sum of Dirac deltas via transporting the mass of $\rho ^0$ inside each *d*-dimensional cube to its center. A cut-off procedure to leave small mass outside a large ball allows us to reduce to a finite number of Dirac deltas in this approximation. Finally, the error produced between smoothed particles and Dirac deltas is obviously of order *h* in the $d_{BL}$ distance.

### Proof of Theorem 3

Since $\rho ^n = F^{t_{n-1},t_n} \# \rho ^{n-1}$ and $\varphi ^n_{h,k} = F^{n-1}_{h,k} \# \varphi ^{n-1}_{h,k}$, we obtain

$$\begin{aligned} \int _{\mathbb R^d} \psi (x) d\rho ^n(x) = \int _{\mathbb R^d} \psi (F^{t_{n-1},t_n}(x)) d\rho ^{n-1}(x), \end{aligned}$$

and

$$\begin{aligned} \int _{\mathbb R^d} \psi (x) d\rho ^n_h(x) = \sum _{k \in \mathbb Z^d}\omega _k\int _{\mathbb R^d} \psi (x) \varphi ^n_{h,k}(x)\,dx = \sum _{k\in \mathbb Z^d}\omega _k\int _{\mathbb R^d} \psi (F^{n-1}_{h,k}(x))\varphi ^{n-1}_{h,k}(x)\,dx, \end{aligned}$$

for $\psi \in \mathcal {W}^{1,\infty }(\mathbb R^d)$ with $\Vert \psi \Vert _{\mathcal {W}^{1,\infty }} \le 1$. Thus, we deduce

$$\begin{aligned} \begin{aligned}&\int _{\mathbb R^d} \psi (x) \left( d\rho ^n(x) - d\rho ^n_h(x)\right) \\&\quad = \int _{\mathbb R^d} \psi (F^{t_{n-1},t_n}(x))\left( d\rho ^{n-1}(x) - d\rho ^{n-1}_h(x) \right) \\&\qquad + \sum _{k \in \mathbb Z^d}\omega _k\int _{\mathbb R^d} \left( \psi (F^{t_{n-1},t_n}(x)) - \psi (F^{n-1}_{h,k}(x)) \right) \varphi ^{n-1}_{h,k}(x)\,dx\\&\quad =: (a) + (b). \end{aligned}\end{aligned}$$

Using $||\nabla (\psi \circ F^{t_{n-1},t_n})||_{L^\infty } \le ||(J^{t_{n-1},t_n})^\mathsf{T}||_{L^\infty } ||\nabla \psi ||_{L^\infty }$, it next follows from (2.5) that $|(a)| \le d_{BL}(\rho ^{n-1},\rho ^{n-1}_h) e^{L\Delta t}$ and we estimate (*b*) with

$$\begin{aligned} \begin{aligned} |(b)|&\le \sum _{k\in \mathbb Z^d} |\omega _k| \int _{S^{n-1}_{h,k}} \Big | \psi (F^{t_{n-1},t_n}(x)) - \psi (F^{n-1}_{h,k}(x)) \Big | \varphi ^{n-1}_{h,k}(x)\,dx \\&\le e_F^{n-1} \sum _{k \in \mathbb Z^d}|\omega _k| \int _{S^{n-1}_{h,k}} \varphi ^{n-1}_{h,k}(x)\,dx \le Ce_F^{n-1} \end{aligned} \end{aligned}$$

where the last inequality uses the estimates inside (2.28). This leads to

$$\begin{aligned} d_{BL}(\rho ^n,\rho ^n_h) \le d_{BL}(\rho ^{n-1},\rho ^{n-1}_h) e^{L\Delta t} + C e_F^{n-1} \end{aligned}$$

and using Proposition 7 we obtain

$$\begin{aligned} d_{BL}(\rho ^n,\rho ^n_h) \le d_{BL}(\rho ^{n-1},\rho ^{n-1}_h) e^{C\Delta t} + C\Delta t(h + \Delta t) \end{aligned}$$

with constants independent of $\Delta t$ and *h*. The proof is then completed using Gronwall’s inequality as in Theorem 1. $\square$

## $L^1$ and $L^p$ convergence for singular potentials

In this part, we are interested in $L^p$-convergence between the solution and its approximation allowing for more singular potentials. With this aim, we consider the solutions of the Eq. (1.1) in $L^\infty (0,T; L^\infty (\mathbb R^d) \cap \mathcal {W}^{1,1}(\mathbb R^d) \cap \mathcal {W}^{1,p}(\mathbb R^d))$ with $1 \le p \le \infty$ to be determined depending on the singularity of the potential. Since we are dealing with both attractive and repulsive potentials, we can only expect local in time existence and uniqueness of solutions as in [15, 24]. In those references, a local in time well-posedness theory in $L^\infty (0,T; L^1(\mathbb R^d) \cap L^{p}(\mathbb R^d))$ was developed under suitable assumptions on the potentials. The solutions are constructed by characteristics since the velocity fields are still Lipschitz continuous in *x*. However, to prove convergence rates we need more regularity on the solutions. For the existence of solutions to (1.1) in $L^\infty (0,T; L^\infty (\mathbb R^d) \cap \mathcal {W}^{1,1}(\mathbb R^d) \cap \mathcal {W}^{1,p}(\mathbb R^d))$, we provide a priori estimates in “Appendix A”, Proposition 10. These estimates combined with the existing literature [15, 24] show the well-posedness of solutions in the desired class. In our presentation we will follow the setting of local existence introduced in [24].

Let us remind the set of hypotheses on the interaction potential called singular potentials in the introduction. We assume that there exists $\tilde{L}>0$ such that

5.1 $$\begin{aligned} | \nabla W(x)|\le \frac{\tilde{L}}{|x|^{\alpha }} \quad \text {and} \quad | D^2 W(x)|\le \frac{\tilde{L}}{|x|^{1+\alpha }} \quad \text{ with } \quad 0 \le \alpha < d-1, \end{aligned}$$

and for $$- 1 \le \alpha < 0$$

5.2 $$\begin{aligned} |\nabla W(x)| \le \tilde{L}\min \left\{ \frac{1}{|x|^\alpha }, 1\right\} \quad \text{ and } \quad |D^2 W(x)| \le \frac{\tilde{L}}{|x|^{1 + \alpha }}. \end{aligned}$$

In particular, singular potentials satisfy $\nabla W \in \mathcal {W}^{1,q}_\mathrm{loc}(\mathbb R^d)$ for all $1 \le q < \frac{d}{\alpha +1}$. Note that (5.1) implies (see [24, 55])

5.3 $$\begin{aligned} |\nabla W(x)-\nabla W(y)| \le \frac{C|x-y|}{\min (|x|,|y|)^{\alpha +1}}. \end{aligned}$$

We remind the reader that these assumptions are enough to guarantee that the velocity fields are bounded and Lipschitz continuous with respect to *x* locally in time for densities in $(L^1\cap L^p)(\mathbb R^d)$ where *p* is the conjugate exponent of *q*. Note that $q = p' < \frac{d}{\alpha + 1}$ is equivalent to $\alpha < -1 + \frac{d}{p'}$, giving us the condition on the initial data for the well-posedness theory. Indeed, it follows from (5.1) that

5.4 $$\begin{aligned} \Vert D u(t,\cdot )\Vert _{L^\infty }&\le \int _{\mathbb R^d} |D^2 W(x-y)| \rho (y)\,dy \le \int _{\mathbb R^d} \frac{\tilde{L}\rho (y)}{|x-y|^{\alpha +1}}\,dy \nonumber \\&\le \left( \int _{|x-y| \ge 1} + \int _{|x-y| \le 1} \right) \frac{\tilde{L}\rho (y)}{|x-y|^{\alpha +1}}\,dy \le C (\Vert \rho (t,\cdot )\Vert _{L^1} + \Vert \rho (t,\cdot )\Vert _{L^p}), \end{aligned}$$

for some constant *C* depending on $\tilde{L}$, *q* and *d*, and a similar estimate holds for *u* using (5.2) and the fact that $\nabla W$ is bounded away from the origin.

Let $T^*$ be the maximal time of existence of weak solutions $\rho \in L^\infty (0,T;(L^1\cap L^p)(\mathbb R^d))$ with $T<T^*$ constructed in [24]. Additional regularity will be needed on these solutions ensured by Proposition 10 of “Appendix A” under suitable initial data assumptions. In this section we consider $T < T^*$, and we denote again $t_n = n\Delta t$ with $0\le n\le N$ and $\Delta t = T/N$ for some given positive integer *N*. We introduce the following notations:

$$\begin{aligned} \begin{aligned} \Vert \cdot \Vert&:=\Vert \cdot \Vert _{L^1}+ \Vert \cdot \Vert _{L^p}, \quad \Gamma ^n_h&:= \Vert \rho ^n - \rho ^n_h\Vert , \quad \text{ and } \quad \widetilde{\Gamma ^n_h} := \sup _{0 \le m \le n}\Gamma ^m_h. \end{aligned} \end{aligned}$$

As for the convergence analysis, we point out that the proof of Sect. 3 cannot be directly applied. Indeed, it is not obvious to obtain an a piori bound on

$$\begin{aligned} \displaystyle \sup _{0\le n \le N} \Vert \rho _h^n\Vert _{L^p} \end{aligned}$$

uniformly in *h* and $\Delta t$, which we need to estimate $(\nabla W * \rho _h^n)$ and $(D ^2 W * \rho _h^n)$. In order to do that, we will prove by induction that there is some $h_* > 0$ for which

$$\begin{aligned} \sup _{0< h \le h_*}\, \widetilde{\Gamma ^N_h} = \sup _{0 < h \le h_*}\, \sup _{0\le n \le N} \Gamma ^n_h \le 1. \end{aligned}$$

We remind the reader that our error analysis between exact and approximated solutions for singular potentials requires non-negative weights for the particles, and this imposes us to give higher regularity on the initial data $\rho ^0 \in \mathcal {W}^{2,p}(\mathbb R^d)$, see Proposition 1. Using the results in [24] and “Appendix A”, we can obtain the existence and uniqueness of a solution $\rho \in L^\infty (0,T;(L^1\cap W^{2,p})(\mathbb R^d))$. However, in the next results we need less regularity in the solutions than on the initial data. Therefore, we prefer to keep both the assumptions stating the needed properties on the solution $\rho$ and the initial data $\rho ^0$ to emphasize this fact.

Under the (induction) assumption that $\widetilde{\Gamma ^n_h}$ is bounded uniformly in *h* and $\Delta t$, we can derive the following estimates.

### Lemma 5

If $M > 0$ and $n \le N$ are such that $\widetilde{\Gamma ^n_h} \le M$, and if the solution to (1.1) satisfies $\rho \in L^\infty (0,T;(L^1\cap L^p)(\mathbb R^d))$, then we have

$$\begin{aligned} \sup _{0 \le m \le n}\Vert \rho ^m_h\Vert \le C_M \quad \text{ and } \quad \sup _{0 \le m \le n}\left( \sup _{x \in \tilde{S}^m_{h,k}} |x-x_k^m|\right) \le C_M(h+\Delta t), \end{aligned}$$

with a constant $C_M$ depending on *M* but not on *h* and $\Delta t$.

### Proof

A straightforward computation yields

$$\begin{aligned} \sup _{0 \le m \le n}\Vert \rho ^m_h\Vert \le \widetilde{\Gamma ^n_h} + \sup _{0 \le t \le T}\Vert \rho (t)\Vert \le C_M. \end{aligned}$$

In a similar way to (5.4), we also bound products like $||D^{(i)} W * \rho ^m_h||_{\mathrm L^{\infty }}$ by $C_W \Vert \rho ^m_h\Vert$ with $C_W = \max ( ||D^{(i)}W||_{L^q(B(0,1))}, ||D^{(i)} W ||_{L^\infty (\mathbb R^d \setminus B(0,1))})$, $i\in \{1,2\}$, from which we derive estimates similar to those of Lemma 2. In particular, following the proof of Lemma 3 we find that for $x \in S^m_{h,k}$,

5.5 $$\begin{aligned} |x - x^m_k| \le \tilde{L} h |(D^m_k)^{-1}| \le \tilde{L} h \exp \left( \Delta t \sum _{l=0}^{m-1} \left|(D^2 W * \rho ^l_h)(x_k^l)\right|\right) \le C_Mh, \end{aligned}$$

and for $x \in \tilde{S}^m_{h,k}$ we find $|x - x^m_k| \le C_M(h+\Delta t)$. Note that this latter estimate involves bounding (5.5) on $S^{m+1}_{h,k}$ which only requires the norm $||\rho ^l_h||$ for $l \le m$, so that the resulting estimate indeed involves a constant depending on *M*. $\square$

We next give the estimates of $u(\tau ,F^{t_m,\tau })-u_k^m$ for $\tau \in [t_{m},t_{m+1}]$ and ${\tilde{\xi }}_m(D^2 W)$ for $0 \le m \le n-1$. The proof can be obtained by using similar arguments as in Proposition 5 with the help of Lemma 5 and a second-order estimate provided either by Proposition 2 or by a standard $L^p$ error estimate as described in Proposition 1. We omit its proof, but point out that the crucial point is the smoothness assumptions (5.3) on the singular potential and the Lipschitz bound (5.4) on the velocity field.

### Lemma 6

If $M > 0$ and $n \le N$ are such that $\widetilde{\Gamma ^n_h} \le M$, and if the solution $\rho \in L^\infty (0,T; \mathcal {W}^{1,1}(\mathbb R^d) \cap \mathcal {W}^{1,p}(\mathbb R^d))$ to (1.1) with initial data $\rho ^0 \in \mathcal {W}^{2,p}(\mathbb R^d)$, then we have

$$\begin{aligned} \sup _{\tau \in [t_{m},t_{m+1}]} | u(\tau ,F^{t_m,\tau }(x_k^m))-u_k^m | \le C_M\left( h^2 +\Delta t +\bar{e}_F^n \right) \end{aligned}$$

and

$$\begin{aligned} {\tilde{\xi }}_m(D^2 W) \le C_M\left( h + \Delta t + \Gamma ^m_h\right) \end{aligned}$$

for $0 \le m \le n$ and $\Delta t$ small enough, with constants $C_M$ depending on *M* but not on *h* and $\Delta t$.

We can also adapt the proof of Corollary 1, Lemma 6, and Proposition 6 to obtain the following result.

### Lemma 7

If $M > 0$ and $n \le N$ are such that $\widetilde{\Gamma ^n_h} \le M$, and if the solution $\rho \in L^\infty (0,T; \mathcal {W}^{1,1}(\mathbb R^d) \cap \mathcal {W}^{1,p}(\mathbb R^d))$ to (1.1) with initial data $\rho ^0 \in \mathcal {W}^{2,p}(\mathbb R^d)$, then we have

$$\begin{aligned} e_j^m \le C_M \Delta t(h + \Delta t + \Gamma ^m_h) \end{aligned}$$

and

5.6 $$\begin{aligned} \tilde{e}_F^m \le C_M \Delta t\left( h^2 + \Delta t + \overline{e_F}^m + (h+\Delta t)\Gamma ^m_h \right) \end{aligned}$$

for $0 \le m \le n$ and $\Delta t$ small enough, with constants $C_M$ depending on *M* but not on *h* and $\Delta t$.

We finally connect the errors to the $L^1\cap L^p$ bounds on the densities.

### Lemma 8

If $M > 0$ and $n \le N$ are such that $\widetilde{\Gamma ^n_h} \le M$, and if the solution $\rho \in L^\infty (0,T; \mathcal {W}^{1,1}(\mathbb R^d) \cap \mathcal {W}^{1,p}(\mathbb R^d))$ to (1.1) with initial data $\rho ^0 \in \mathcal {W}^{2,p}(\mathbb R^d)$, then we have

$$\begin{aligned} \overline{e_F}^{m+1} \le C_M( h^2 + \Delta t + h \widetilde{\Gamma ^{m}_h}), \end{aligned}$$

and

$$\begin{aligned} \tilde{e}_F^m \le C_M \Delta t\left( h^2 + \Delta t + (h+\Delta t) \widetilde{\Gamma ^m_h} \right) \end{aligned}$$

for all $0\le m \le n$ and $\Delta t$ small enough, with constants $C_M$ depending on *M* but not on *h* and $\Delta t$.

### Proof

Since Lemma 4 only relies on the Lipschitz smoothness of the exact flow, we have

$$\begin{aligned} \overline{e_F}^{m+1} \le e^{C\Delta t}\overline{e_F}^m + \tilde{e}_F^m \end{aligned}$$

for all *m*. Then from (5.6) we derive

$$\begin{aligned} \overline{e_F}^{m+1} \le e^{(C+C_M)\Delta t}\overline{e_F}^m + C_M \Delta t\left( h^2 + \Delta t + h\Gamma ^m_h \right) \end{aligned}$$

for $m \le n$, so that Gronwall’s inequality (together with $\widetilde{\Gamma ^m_h} = \max _{m'\le m} \Gamma ^{m'}_h$) yields

$$\begin{aligned} \overline{e_F}^{m+1} \le C_M(h^2 + \Delta t + h\widetilde{\Gamma ^m_h}) \end{aligned}$$

due to $\overline{e_F}^0 = 0$. Using this together with (5.6) completes the proof. $\square$

We are now in a position to show the uniform $L^1 \cap L^p$ bounds on the density.

### Proposition 8

Assume that the interaction potential *W* is singular in the sense of (5.1) and (5.2), and let $\rho$ be a solution to the Eq. (1.1) up to time $T>0$, such that $\rho \in L^\infty (0,T;(\mathcal {W}^{1,1} \cap \mathcal {W}^{1,p} \cap L^\infty )(\mathbb R^d))$ with initial data $\rho ^0 \in \mathcal {W}^{2,p}(\mathbb R^d)$, $-1 \le \alpha < -1 + d/p'$, and $1 < p \le \infty$. Assume in addition that $\Delta t \lesssim h^2 \le 1$. Then for all $M > 0$, there exists $h_*(M) > 0$ such that

$$\begin{aligned} \sup _{0 < h \le h_*(M)} \, \sup _{0 \le n \le N} \Gamma ^n_h \le M. \end{aligned}$$

### Proof

We use an induction argument on *n*. Since $\widetilde{\Gamma ^0_h} = \Gamma ^0_h \lesssim h^2$, clearly there exists $h_0(M)$ such that $\Gamma ^0_h \le M$ for all $h < h_0(M)$. We then assume that $n < N$ and $h_n(M) > 0$ are such that

$$\begin{aligned} \sup _{0 < h \le h_n(M)} \widetilde{\Gamma ^n_h} \le M. \end{aligned}$$

For the remaining of the proof we then consider $m \le n$ and $h \le h_n(M)$. In particular, we observe that the Lemmas above can be used with this value of *M*. Decomposing the error as in Theorem 1, we write

$$\begin{aligned} \begin{aligned}&\rho ^{m+1}(y) - \rho _h^{m+1}(y)\\&\quad = \underbrace{ \left[ \rho \left( t_{m}, F^{t_{m+1},t_{m}}(y)\right) - \rho _h^{m}\left( F^{t_{m+1},t_{m}}(y)\right) \right] j^{t_{m+1},t_{m}}(y) }_{A_{m+1}(y)} \\&\qquad + \underbrace{ \sum _{k\in \mathbb Z^d} \frac{\omega _k }{h_k^m} \varphi \left( \frac{D_k^m}{h}\left( F^{t_{m+1},t_{m}}(y)- x_k^{m}\right) \right) \left[ j^{t_{m+1},t_{m}}(y) - \frac{1}{j_{k}^{m}} \right] }_{B_{m+1}(y)} \\&\qquad + \underbrace{\sum _{k\in \mathbb Z^d} \frac{\omega _k }{h_k^{m+1}} \left[ \varphi \left( \frac{D_k^{m}}{h}(F^{t_{m+1},t_{m}}(y)- x_k^{m} ) \right) - \varphi \left( \frac{D_k^{m+1}}{h} (y- x_k^{m+1}) \right) \right] }_{C_{m+1}(y)}. \end{aligned} \end{aligned}$$

Using arguments similar than in Theorem 1 we find

$$\begin{aligned} \Vert A_{m+1}\Vert _{L^p} \le e^{C \Delta t} \Vert \rho _h^m-\rho ^m\Vert _{L^p} \quad \text{ and } \quad \Vert B_{m+1}\Vert _{L^p} \le Ce_j^{m}\Vert \rho ^{m}_h\Vert _{L^p} \le C_M e_j^m. \end{aligned}$$

For the estimate of $C_{m+1}(y)$, we use the interpolation inequality and the estimates in Theorems 1 and 2 to get

$$\begin{aligned} \begin{aligned} \Vert C_{m+1}\Vert _{L^p}&\le \Vert C_{m+1}\Vert _{L^1}^{1/p}\Vert C_{m+1}\Vert _{L^\infty }^{1/q} \\&\le C_M\frac{(\tilde{e}_F^{m})^{1/p}}{h^{1/p}} \left( 1 + \frac{\overline{e_F}^{m} + \overline{e_F}^{m+1}}{h}\right) ^{d/q}\frac{(\tilde{e}_F^{m})^{1/q}}{h^{1/q}} \\&= C_M\left( 1 + \frac{\overline{e_F}^{m} + \overline{e_F}^{m+1}}{h}\right) ^{d/q}\frac{\tilde{e}_F^{m}}{h}. \end{aligned} \end{aligned}$$

Using Lemma 8 and the fact that $\widetilde{\Gamma ^m_h} \le M$ and $\Delta t \lesssim h$ we find that both $\overline{e_F}^{m}$ and $\overline{e_F}^{m+1}$ are bounded by $C_M h$, thus

$$\begin{aligned} \Vert C_{m+1}\Vert _{L^p} \le C_M \frac{\tilde{e}_F^{m}}{h}, \end{aligned}$$

and the above estimates yield

$$\begin{aligned} \Vert \rho ^{m+1} - \rho _h^{m+1}\Vert _{L^p} \le e^{C\Delta t}\Vert \rho ^m - \rho _h^m\Vert _{L^p} + C_M \left(e_j^{m} + \frac{\tilde{e}_F^{m}}{h}\right). \end{aligned}$$

We also observe that in the proof of Theorem 1, all the steps leading to the estimate

$$\begin{aligned} \theta _{m+1} \le \theta _m + C_M \left(e_j^{m} + \frac{\tilde{e}_F^{m}}{h}\right) \end{aligned}$$

(where we remind that $\theta _m = \Vert \rho ^{m} - \rho _h^{m}\Vert _{L^1}$) are valid in the case of singular potentials. This yields

$$\begin{aligned} \Gamma ^{m+1}_h \le e^{C\Delta t}\Gamma ^m_h + C_M\left( e_j^{m} + \frac{\tilde{e}_F^{m}}{h}\right). \end{aligned}$$

On the other hand, it follows from Lemmas 7 and 8 that

$$\begin{aligned} e_j^m \le C_M \Delta t(h + \Delta t + \Gamma ^m_h) \le C_M\Delta t\left( h + \Gamma ^m_h\right), \end{aligned}$$

and

$$\begin{aligned} \frac{\tilde{e}_F^{m}}{h} \le C_M\Delta t \left(h + \frac{\Delta t}{h} + \left(1 + \frac{\Delta t}{h}\right)\widetilde{\Gamma ^m_h}\right) \le C_M\Delta t (h + \widetilde{\Gamma ^m_h}), \end{aligned}$$

where we used the assumption $\Delta t \lesssim h^2$. Thus we find

$$\begin{aligned} \widetilde{\Gamma ^{m+1}_h} \le e^{(C+C_M)\Delta t}\widetilde{\Gamma ^m_h} + C_Mh\Delta t. \end{aligned}$$

Since this is valid for all $m\le n$, it follows from Gronwall’s lemma that $\widetilde{\Gamma ^{n+1}_h} \le C_Mh$ holds for some constant $C_M > 0$. We remind the reader that $C_M$ is the generic constant depending on *M* but independent of *h* and $\Delta t$. In particular, setting $h_{n+1}(M) := \min (h_n(M), M/C_M)$ allows to write

$$\begin{aligned} \sup _{0 < h \le h_{n+1}(M)} \widetilde{\Gamma ^{n+1}_h} \le M. \end{aligned}$$

This ends the induction argument and the proof, by taking $h_*(M) = h_N(M)$. $\square$

Putting together all the results in this section, we obtain the main convergence result in $(L^1 \cap L^p)(\mathbb R^d)$. We note that, as above, the condition on the time step is a result of the low order time discretization (see Remark 3).

### Theorem 4

Assume that the interaction potential *W* is singular in the sense of (5.1) and (5.2), and let $\rho$ be a solution to the Eq. (1.1) up to time $T>0$, such that $\rho \in L^\infty (0,T;(\mathcal {W}^{1,1} \cap \mathcal {W}^{1,p} \cap L^\infty )(\mathbb R^d))$ with initial data $\rho ^0 \in \mathcal {W}^{2,p}(\mathbb R^d)$, $-1 \le \alpha < -1 + d/p'$, and $1 < p \le \infty$. Assume in addition that $\Delta t \lesssim h^2 \le 1$. Then

$$\begin{aligned} \sup _{0 < h \le h_*} \sup _{0\le n \le N} \Vert \rho _h^n-\rho ^n\Vert \le Ch \end{aligned}$$

holds with $h_* = h_*(1)$ given by Proposition 8 and a constant *C* independent of *h* and $\Delta t$.

## Numerical results

We will present in this Section some numerical examples in one dimension, with different interaction potentials and initial densities to showcase some of the features already observed in numerical and theoretical analysis of the aggregation equation (1.1) in [4, 11, 14, 49, 50, 57]. In this way, we first validate our numerical implementation in order to explore some less-known properties about the behavior of its solutions in one dimension. A further more complete numerical study in 2D of this method will be reported elsewhere. These examples already show the wide range of different behaviors of solutions to the aggregation equation.

### Numerical method: computation of the velocity field

In (2.31) we see that we need to compute at each time step the velocity field on the particles,

6.1 $$\begin{aligned} u_k^n := - \nabla W * \rho _h^n(x_k^n) = - \int _{\mathbb R^d} \nabla W(x_k^n-y) \rho _h^n(y)dy \qquad \text{ for } k \in \mathbb Z^d. \end{aligned}$$

In practice these products need to be approximated, and several strategies can be used for that purpose in one dimension or more. The simplest one actually consists of replacing the linearly-transformed particle shapes involved in the density (2.27) by a fixed blob shape $\zeta _\varepsilon = \varepsilon ^{-d}\zeta (\frac{\cdot }{\varepsilon })$, leading to

$$\begin{aligned} u_{\mathrm{blob},k}^n = - \sum _{k'\in \mathbb Z^d} \omega _{k'} \nabla W_\varepsilon (x_k^n - x_{k'}^n) \end{aligned}$$

where $W_\varepsilon = W * \zeta _\varepsilon$ is the corresponding smoothed kernel and $\omega _{k'}$ is the weight of the particle $k'$. Here the velocities are computed as in a standard particle method, however the approximated density (2.27) is more accurate as it still involves the LTP shapes (2.23). If the latter is used to occasionally resample new weighted particles, as successively carried out e.g. in [20] for the Vlasov-Poisson system, the resulting scheme effectively differs from a standard one.

A second strategy consists of computing first the values $(\rho _{h,j}^n)_{j \in {\mathcal G}}$ on a grid $(\chi _j)_{j\in {\mathcal G}}$ and then using a classical quadrature formula for the product (6.1) on each particle. This leads to an approximed velocity of the form

$$\begin{aligned} u_{\mathrm{quad},k}^n = - \sum _{j\in {\mathcal G}} \alpha _j \nabla W(x_k^n-\chi _j) \rho _{h,j}^n. \end{aligned}$$

A variant of this second approach would be to compute the velocity field on the grid,

$$\begin{aligned} u_{\mathrm{grid}, i}^n = - \sum _{j\in {\mathcal G}} \alpha _j \nabla W(\chi _i-\chi _j) \rho _{h,j}^n, \quad \text{ for } i\in {\mathcal G}, \end{aligned}$$

which can then be interpolated on the particles. We note that discrete convolution products can be computed using standard library routines based on the fast Fourier transform, which effectively reduces the computational cost of this approach.

Obviously, the cost of the above strategies depends on the numbers of particles and grid points. The first one scales like $N_\mathrm{parts}^2$, the second one like $N_\mathrm{parts} N_\mathrm{grid}$ and the third one like $N_\mathrm{grid}\log (N_\mathrm{grid})$. For our simulations we have tried these three strategies (with $N_\mathrm{grid} \sim N_\mathrm{parts}$) and have observed no significant differences in the resulting densities.

It is worth mentioning that the vortex method for the 2D Euler equations is studied in [72], where the vorticity is approximated by a piecewise interpolation polynomial on a triangulation of the vortices and the vertices of the triangulation move with the fluid velocity. In our case, the particles could have a piecewise affine shape, but their supports can intersect as mentioned before. Thus our method of computing the velocity fields in general cannot be reduced to a *P*1-finite element discretization of the velocity fields as in [72], see also [37] for other remeshed particle methods and general convergence proofs.

### Numerical method: validation and comparison to classical particle methods

We have implemented the numerical method described in Sect. 2.2 using Python. We use different initial conditions depending on the behaviors we would like to show. Specifically, we consider as initial densities

6.2 $$\begin{aligned} \rho ^0_1(x)= & {} (e^{-30(x-0.5)^2}+ 2e^{-50(x+0.3)^2}){\mathbb {1}}_{[-1,1]}(x), \end{aligned}$$

6.3 $$\begin{aligned} \rho ^0_2(x)= & {} {\mathbb {1}}_{[-1,1]}(x), \end{aligned}$$

6.4 $$\begin{aligned} \rho ^0_3(x)= & {} (1-x^2)^{20} {\mathbb {1}}_{[-1,1]}(x), \end{aligned}$$

6.5 $$\begin{aligned} \rho ^0_4(x)= & {} e^{(x^2-1)^{-1}} {\mathbb {1}}_{[-1,1]}(x), \end{aligned}$$

in order to have asymmetric, discontinuous symmetric and compactly supported smooth initial data. Shape functions for the particle method are here B3-splines given by (2.13). We first examine the validation of our code by comparison of the numerical solution and the exact solution of (1.1) with $W(x)= x^2$. Due to the conservation of the center of mass,

$$\begin{aligned} \forall t \ge 0, \qquad \int _\mathbb Rx\rho (t,x)dx= \int _\mathbb Rx\rho ^0(x)dx:=\lambda , \end{aligned}$$

the solution is explicitly given by the method of characteristics,

6.6 $$\begin{aligned} \rho (t,x)=\rho ^0\left( (x-\lambda )e^{2t}+\lambda \right) e^{2t}. \end{aligned}$$

**Fig. 2:**
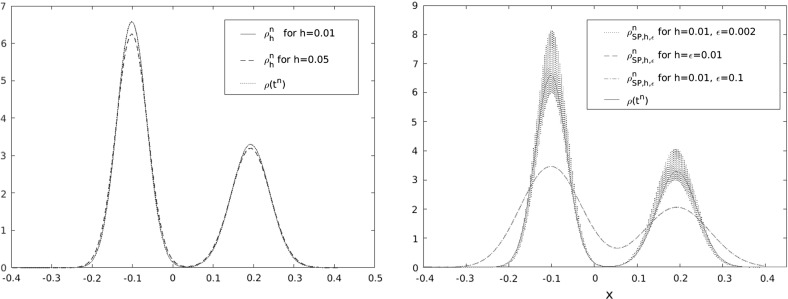
Comparisons for $W(x)=x^2$, at $t=0.5$ with initial data (6.2) and $\Delta t=10^{-4}$. Left: comparison between the exact solution $\rho (t,x)$, see (6.6), and the LTP approximation $\rho _h^n$, for two values of the average particle distance *h*. Right: approximated values $\rho _{SP,h,\epsilon }^n$ obtained with a classical smooth particle (SP) method for $h=0.01$ and different values for the constant particle size $\varepsilon$

**Fig. 3:**
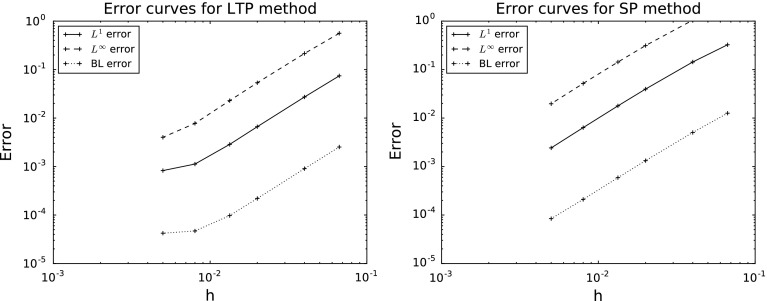
Log–log plot of the $L^1$, $L^{\infty }$ and $d_{BL}$ errors at $t=0.5$ for the LTP (left) and SP (right) methods with different values of $h = \varepsilon$. Here the problem parameters *W*, $\rho ^0$ and the time step $\Delta t$ are the same as in Fig. 2

The left plot of Fig. 2 compares this exact solution with the LTP approximation at time $t=0.5$, both with initial data (6.2), and several error curves are shown in Fig. 3(left), namely $L^1$, $L^{\infty }$ errors and an error curve in the bounded Lipschitz distance $d_{BL}(\rho _h^n,\rho (t^n))$. In dimension 1, this distance is computed according to

$$\begin{aligned} d_{BL}(\rho _1,\rho _2) = \Vert F_1 - F_2 \Vert _1, \quad \text{ where } F_i(x) = \int _{-\infty }^x \rho _i(u)du, \quad i=1,2. \end{aligned}$$

In the right plot of Fig. 2 we compare the exact solution with several approximations using a classical Smooth Particle (SP) method, in which the density is reconstructed with shape functions of uniform size $\varepsilon$,

$$\begin{aligned} \rho _{SP,h,\varepsilon }^n(x)= \sum _{k\in \mathbb Z} \omega _k \frac{1}{\varepsilon } \varphi \left( \frac{x-x_k^n}{\varepsilon } \right) . \end{aligned}$$

One difficulty of such methods lies in the choice of an adequate value for the particle size $\varepsilon$. If this value is too small compared to the average distance *h* between two particles, the reconstructed density will oscillate or even vanish between nearby particles and thus become inaccurate; if $\varepsilon$ is too large the reconstructed density will be too spread out and the results will again lack accuracy, as demonstrated in Fig. 2(right).

**Fig. 4:**
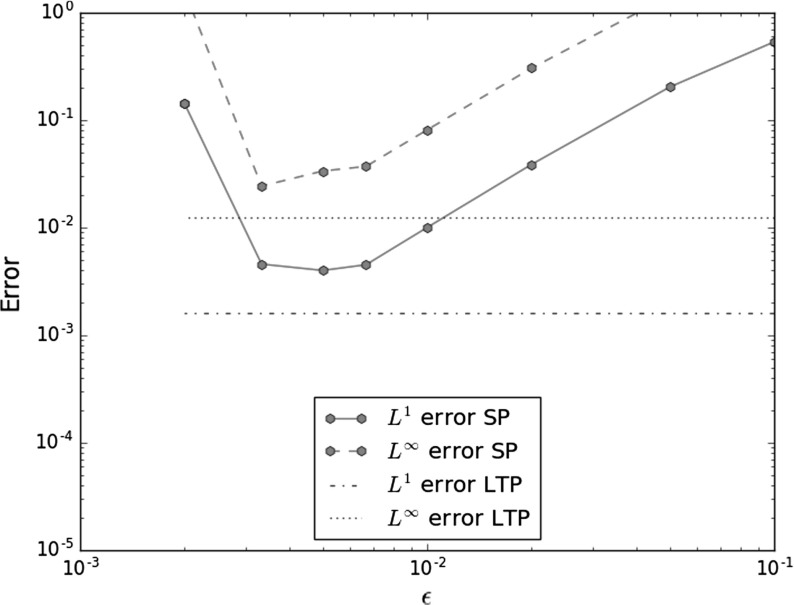
Log–log plot of the $L^1$ and $L^{\infty }$ errors of the LTP and SP methods, with $h = 0.01$ and various values of $\varepsilon$. Here *W*, $\rho ^0$, $\Delta t$ and *t* are the same as in Fig. 2

In Figs. 3 and 4 we further compare the Smooth Particle (SP) and the LTP approximations by showing $L^1$, $L^{\infty }$ and $d_{BL}$ error curves, using several values of *h* and $\varepsilon$. Again the potential is $W(x)=x^2$ and the exact solution is given by (6.6).

**Fig. 5:**
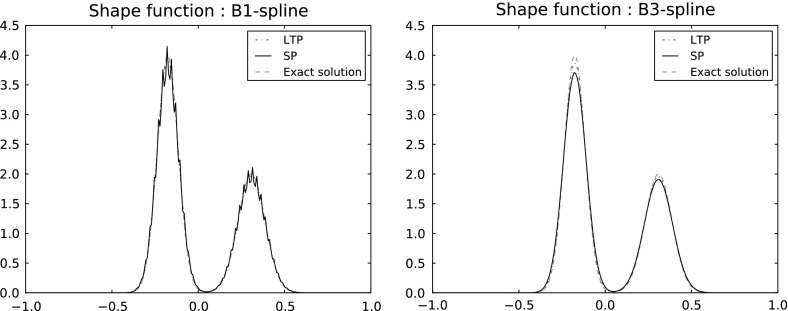
Comparisons between exact, LTP and SP solutions at $t=0.5$. Here $W(x)=x^2/2$, $\rho ^0$ is (6.2), $h=1/25$ and $\Delta t=10^{-3}$. On the left the shape function $\varphi$ is a hat function, and on the right it is a cubic B3-spline

Together with Fig. 2, these error curves show not only the higher accuracy reached by the LTP method but also the sensitivity of the final error with respect to the particle size $\varepsilon$. An interesting feature of the LTP approach is the automatic adaptation of the particle size, and for the cases considered here, Figs. 3 and 4 show that such an approach outperforms any uniform choice of $\varepsilon$. In Fig. 5 some comparisons are shown with B1 and B3 spline shape functions, and again the gain of accuracy reached by the LTP method is clear.

**Fig. 6:**
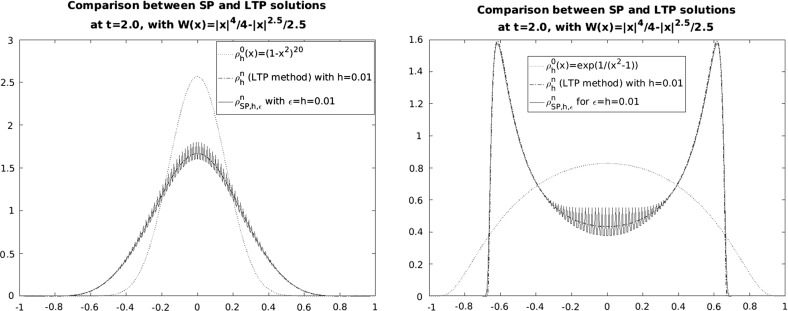
Comparisons of LTP and SP solutions at time $t=2.0$, with $W(x)=x^4/4-|x|^{2.5}/2.5$, $\varepsilon = h=0.01$ and $\Delta t=10^{-2}$. Here $\rho ^0$ is (6.4) on the left and (6.5) on the right. The localization of the oscillations in the SP solutions gives a hint that the optimal particle size is space-dependent. See also Fig. 10 below

A further advantage of the LTP method lies in the time and space adaptation of the particle size. Indeed as particles aggregate, the average particle distance evolves in time and may also depend on the spatial position *x*. In the case of potential $W(x)=x^2$, the explicit expression of the density (6.6) shows that $j^{0,t}(x)=e^{-2t}$, and thus the average distance between two particles decreases exponentially in time. Consequently, the optimal size $\varepsilon$ for reconstruction in classical particle method is not the same during the whole simulation: An evolution in time of $\varepsilon$ may be better adapted. A similar issue may also appear regarding the space dependency. In fact for the potential $W(x)=x^2$ considered in Figs. 2, 3 and 4, the Jacobian determinant $j^{0,t}$ was constant with respect to *x* and all the particles in the LTP method had the same size at a given time *t*. However in general this is not the case: with the potential $W(x)= \frac{x^4}{4}- \frac{|x|^{2.5}}{2.5}$ considered in Figs. 6 and 10 with various initial densities, we see that the Smooth Particle method with $\varepsilon =h$ leads to solutions that seem accurate in some regions but strongly oscillate in some others. And in this case, Fig. 10 (bottom) shows that the size of the LTP particles evolves in space, thus giving a hint that an optimal particle size is indeed space-dependent.

### Numerical simulations: singular potentials and qualitative properties of steady states

**Fig. 7:**
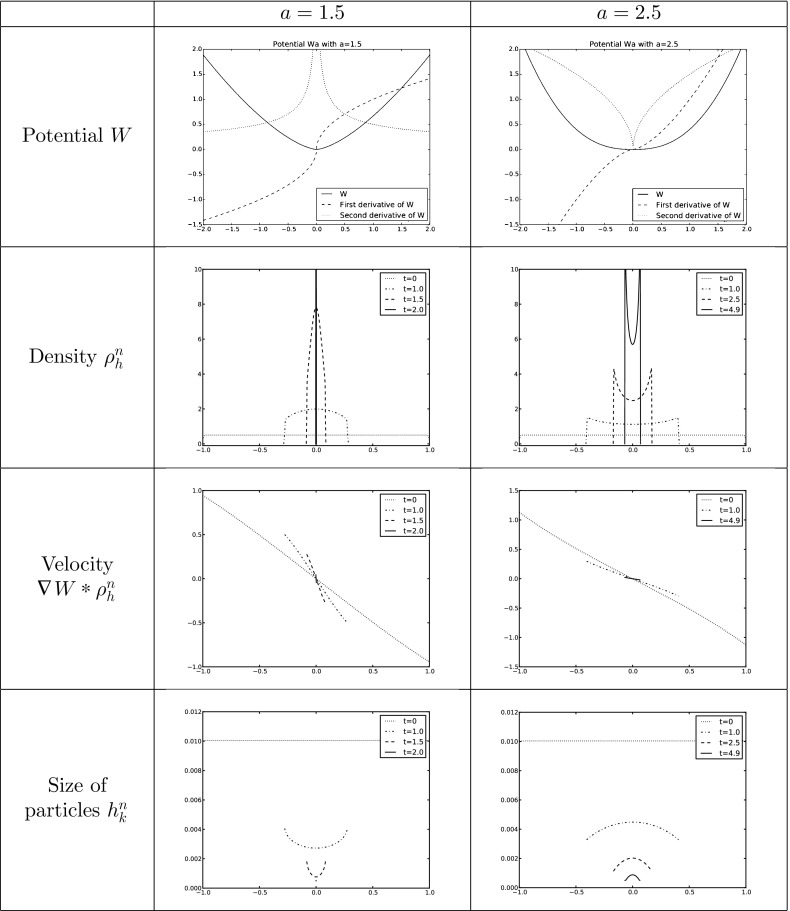
Approximate densities, reconstructed velocities and particles sizes computed by the LTP method for $W(x)=\tfrac{|x|^a}{a}$ with $a=1.5$ or $a=2.5$. Here $h=0.01$, $\Delta t = 0.01$ and $\rho ^0$ is (6.3)

We now take advantage of the method to explore the behavior for other attractive potentials of type $W(x)=\tfrac{|x|^{a}}{a}$, $a>1$. Notice that for $a\ge 2$ the potential is smooth while for $1<a<2$ it is singular once *W* is cut-off at infinity or if the initial data is compactly supported since the effective values of the potential lie on a bounded set and *W* can be cut-off at infinity without changing the solution. Figure 7 presents the numerical results obtained by the LTP method in the case of $a=1.5$ and $a=2.5$. We represent the approximate density $\rho _h^n$, the reconstructed velocity $u_h^n$ and the reconstructed particles sizes $h^n$ using piecewise linear interpolation such that

$$\begin{aligned} u_h^n(x_k^n) = - \nabla W * \rho _h^n(x_k^n) \quad \text{ and } \quad h^n(x_k^n) = h \prod _{m=0}^{n-1} j_k^m. \end{aligned}$$

Potentials and their derivatives are also represented. In both cases, we observe that the density converges to a Dirac mass. Figure 7 also shows that for $a=2.5$, $W'' \in L_{loc}^{\infty }$, no finite-time blow-up in $L^\infty$ appears, opposite to the case $a=1.5$ in agreement with the results proved in [12]. Notice also the different qualitative behavior in their trend to blow-up as studied in [57].

**Fig. 8:**
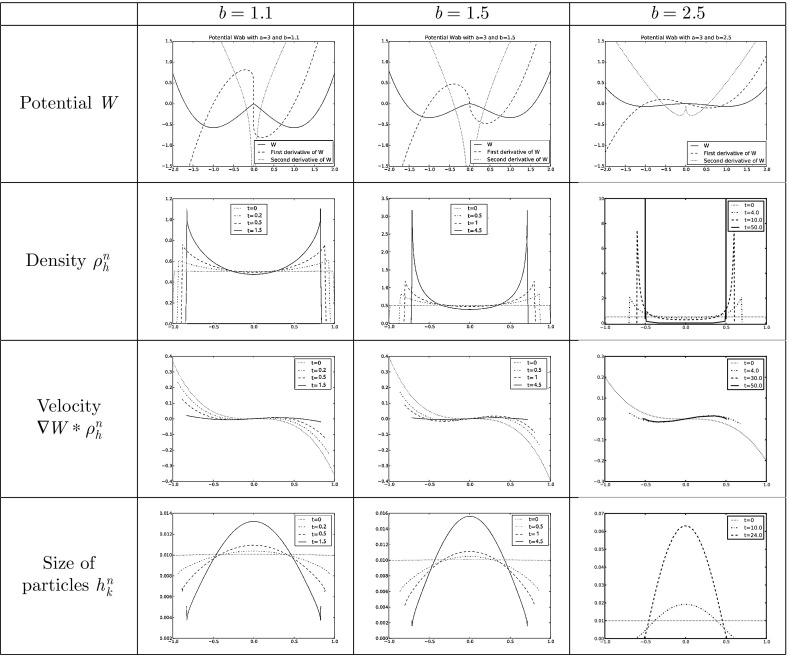
Approximate densities, reconstructed velocities and particles sizes computed by the LTP method for $W(x)=\frac{|x|^a}{a} -\frac{|x|^b}{b}$, with $a=3$ and $b=1.5$ or $b=2.5$. As in Fig. 7, $h=0.01$, $\Delta t = 0.01$ and $\rho ^0$ is (6.3)

Next we further analyze the blow-up behavior by looking at the case of attractive-repulsive potentials $W(x)=\tfrac{|x|^a}{a} -\tfrac{|x|^b}{b}$, $1<b<a$. Notice again that for $b\ge 2$ the potential is smooth while for $1<b<2$ it is singular once *W* is cut-off at infinity or if the initial data is compactly supported as discussed above. Figure 8 presents the approximate density $\rho _h^n$, the reconstructed velocity $u_h^n$ and the particles sizes $h^n$ obtained by the LTP method in the case of the attractive-repulsive potentials with $(a,b)=(3,1.5)$ and $(a,b)=(3,2.5)$. In this case $\rho ^0$ is given by (6.3).

We observe that the long time asymptotics for $b=2.5$ are characterized by the concentration of mass equally onto Dirac deltas at two points in infinite time, while for $b=1.5$ we obtain a convergence in time towards a steady $L^1$ density profile seemingly diverging at the boundary of the support. This last behavior has been reported in several simulations and related problems [11]. However, it has not been rigorously proven yet. Let us point out that the set of stationary states where the interaction potential is analytic in 1D consists of a finite number of Dirac deltas as proven in [49, 50]. This result also holds for $W(x)=\tfrac{|x|^a}{a} -\tfrac{|x|^b}{b}$, $2<b<a$, as it will be reported in [29].

**Fig. 9:**
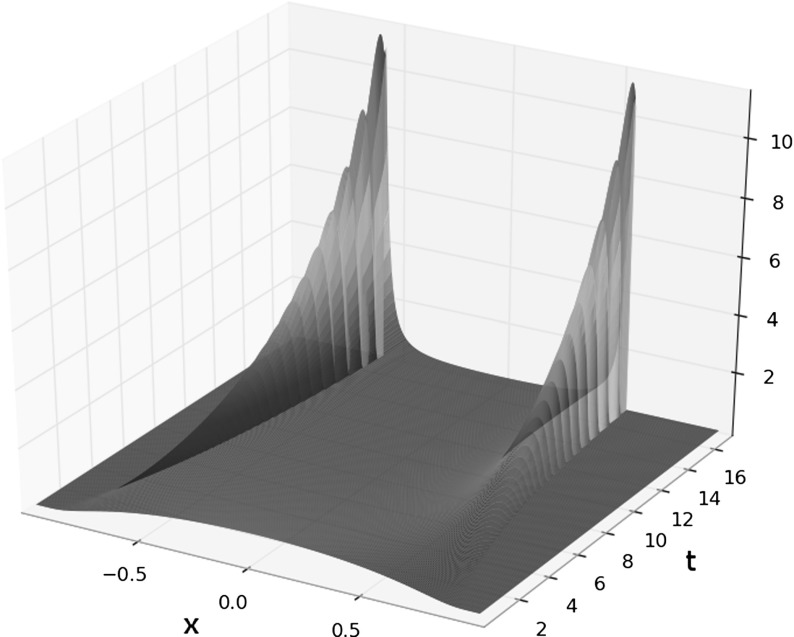
Time evolution of the LTP density $\rho ^n_h$ corresponding to $W(x)=\frac{|x|^a}{a} -\frac{|x|^b}{b}$ with $a=3$ and $b=2.5$, $\rho ^0$ given by (6.3) and $h=0.01$, $\Delta t = 0.01$, as in Fig. 8 (right)

Figure 9 also represents the time evolution of the approximated density for $(a,b)=(3,2.5)$, with $\rho ^0$ given by (6.3). Solutions in the range $2<b<a$ for initial data in $L^1\cap L^\infty$ exist globally in time, see [53]. The numerical evidence shows that all solutions converge towards stationary states consisting of finite number of Dirac Deltas as $t\rightarrow \infty$ in this range.

**Fig. 10:**
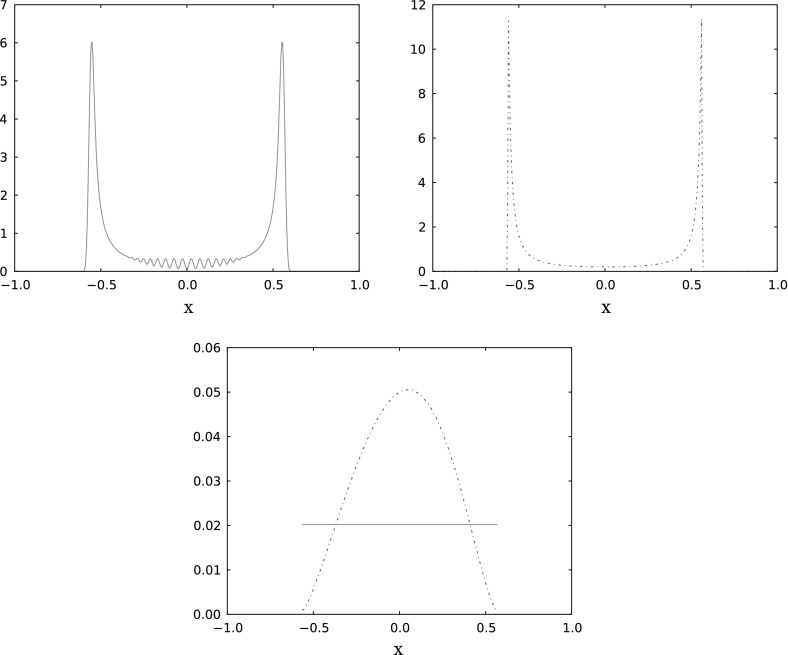
Densities at steady state for $W(x)=\frac{|x|^a}{a} -\frac{|x|^b}{b}$, with $a=4$, $b=2.5$ and $\rho ^0$ given by (6.3). Top left: SP method (solid line), top right: LTP method (dotted line), bottom: particles size (SP and LTP) using $h=0.01$ and $\Delta t = 0.01$

Finally, we show in Fig. 10 the results of the stationary state of the SP method versus the LTP method for the potential $(a,b)=(4,2.5)$ with $N=100$. We observe how the good local adaption of the size of the particles makes our approximation much better with no oscillations with respect to the SP method showing the good performance of the LTP method in this case and its good properties at work. As mentioned in the introduction, vortex-blob type methods have been shown to converge for the aggregation equation (1.1). Convergence estimates in suitable $L^p$ norms have been shown for the velocity fields and the associated characteristics fields, while the density errors have been controlled in suitable $W^{-1,p}$-norms in [13, Th. 3.8]. The error estimates for vortex-blob and SP methods depend as usual on the regularization of particles and the fixed particle size related in a suitable way to get convergence. We have proven that the LTP method has in contrast direct error estimates for the densities in $L^p$ depending on the initial mesh size showing that the local adaptation of the shape has this benefit on the error estimates too.

## Appendix A: A priori estimates on the regularity of solutions

In this part, we deduce a priori estimates on the regularity of Eq. (1.1) that combined with the global/local in time well posedness theory obtained in [15, 24, 45, 53], leads to the existence of solutions with the desired properties to apply the convergence results of previous sections.

As we remind the reader in the introduction and in several places along the text, there are two different well-posedness settings: for smooth and for singular potentials. In both cases under the assumptions on the initial data the velocity fields are continuous in time and Lipschitiz continuous in space. In the smooth potential case, this property holds globally in time leading to unique global in time measure solutions [45, 53]. In the singular potential case, this property holds locally in time only since there exist blowing-up of solutions for fully atractive potentials, see [12, 15, 24]. In both cases, the flow map associated to the velocity field is well-defined and solutions are obtained by pushing forward the initial data through the flow map.

In this Appendix, we present first a global-in-time propagation of regularity result in the smooth potential case adapted to our hypotheses on the convergence results. On the other hand, we show a local-in-time propagation of regularity result in the singular potential case.

### Proposition 9

Assume that the interaction potential *W* satisfies $\nabla W \in \mathcal {W}^{1,\infty }(\mathbb R^d)$. Let $T >0$ be given and $\rho$ be the unique weak solution to the system (1.1) with initial data $\rho ^0 \in \mathcal {W}_+^{1,1}(\mathbb R^d)$ obtained in [45, 53], then

$$\begin{aligned} \sup _{0 \le t \le T}\Vert \rho (t,\cdot )\Vert _{\mathcal {W}_+^{1,1}} \le C, \end{aligned}$$

where *C* is a positive constant depending only on *T*, *L*, and $\Vert \rho ^0\Vert _{\mathcal {W}_+^{1,1}}$. Furthermore, if we assume that the initial data $\rho ^0 \in (L^\infty \cap \mathcal {W}_+^{1,1})(\mathbb R^d)$, then

$$\begin{aligned} \sup _{0 \le t \le T}\Vert \rho (t,\cdot )\Vert _{L^\infty \cap \mathcal {W}^{1,1}_+} \le C, \end{aligned}$$

where *C* is a positive constant depending only on *T*, *L*, and $\Vert \rho ^0\Vert _{L^\infty \cap \mathcal {W}_+^{1,1}}$.

### Proof

It follows from the conservation of mass and our assumption on the initial data $\rho ^0$ that

$$\begin{aligned} \int _{\mathbb R^d} \rho (t,x) \,dx = \int _{\mathbb R^d} \rho ^0(x) \,dx = 1. \end{aligned}$$

For the estimate of $\Vert \rho \Vert _{L^\infty (0,T;\dot{\mathcal {W}}^{1,1})}$, we take $\nabla$ to (1.1) to get

A.1 $$\begin{aligned} \begin{aligned}&\partial _t \nabla \rho (t,x) + D^2 \rho (t,x) u(t,x) + \nabla u(t,x) \nabla \rho (t,x) \\&\quad + \nabla (\nabla \cdot u(t,x)) \rho (t,x) + \nabla \cdot u(t,x) \nabla \rho (t,x) = 0. \end{aligned} \end{aligned}$$

We next multiply (A.1) by $\nabla \rho (t,x)/|\nabla \rho (t,x)|$ to obtain

A.2 $$\begin{aligned} \begin{aligned}&\partial _t |\nabla \rho | + u\cdot \nabla |\nabla \rho (t,x)| + \nabla \cdot u|\nabla \rho (t,x)| \\&\quad =- \nabla u \nabla \rho \cdot \frac{\nabla \rho }{|\nabla \rho |} - \nabla (\nabla \cdot u) \rho \cdot \frac{\nabla \rho }{|\nabla \rho |}, \end{aligned} \end{aligned}$$

due to the symmetry of $D^2 \rho$. By integrating (A.2) over $\mathbb R^d$ and using integration by parts, we deduce

A.3 $$\begin{aligned} \begin{aligned} \frac{d}{d t}\int _{\mathbb R^d}|\nabla \rho |\,dx =&-\int _{\mathbb R^d} \nabla u \nabla \rho \cdot \frac{\nabla \rho }{|\nabla \rho |}\,dx \\&- \int _{\mathbb R^d} \nabla (\nabla \cdot u) \rho \cdot \frac{\nabla \rho }{|\nabla \rho |}\,dx\le 2L |\nabla \rho |\,dx, \end{aligned} \end{aligned}$$

where we used $\Vert \nabla u(t,x)\Vert _{L^\infty } \le \Vert \nabla W\Vert _{\mathcal {W}^{1,\infty }} =L$ and

$$\begin{aligned} \Vert \nabla (\nabla \cdot u)\Vert _{L^\infty } \le L\int _{\mathbb R^d} |\nabla \rho |\,dx. \end{aligned}$$

Thus we have

$$\begin{aligned} \sup _{0 \le t \le T}\Vert \nabla \rho (t,\cdot )\Vert _{L^1} \le \Vert \nabla \rho ^0\Vert _{L^1}\exp \left(2LT\right). \end{aligned}$$

Finally, we estimate $\Vert \rho \Vert _{L^\infty }$. For this, we recall that the flow map $F^{0,t}(x)$ satisfies

$$\begin{aligned} \frac{d F^{0,t}(x)}{dt} = u(t, F^{0,t}(x)) \quad \text{ with } \quad F^{0,0}(x) = x. \end{aligned}$$

Using that $\rho (t)= F^{0,t}\#\rho ^0$, we can write

$$\begin{aligned} \frac{\partial }{\partial t}\rho (t,F^{0,t}(x)) = -\nabla \cdot u(t,F^{0,t}(x))\rho (t,F^{0,t}(x)), \end{aligned}$$

and this yields

$$\begin{aligned} \rho (t,F^{0,t}(x)) = \rho ^0(x)\exp \left( -\int _0^t \nabla \cdot u(s,F^{0,s}(x))\,ds\right) . \end{aligned}$$

Since $u \in \mathcal {W}^{1,\infty }(\mathbb R^d)$, we obtain

A.4 $$\begin{aligned} \sup _{0 \le t \le T}\Vert \rho (t,\cdot )\Vert _{L^\infty } \le \Vert \rho ^0\Vert _{L^\infty }\exp \left( LT\right) . \end{aligned}$$

This completes the proof. $\square$

### Remark 5

If we further assume that $\rho ^0 \in \mathcal {W}_+^{1,\infty }(\mathbb R^d)$, we have

$$\begin{aligned} \sup _{0 \le t \le T}\Vert \rho (t,\cdot )\Vert _{\mathcal {W}^{1,\infty }} \le C, \end{aligned}$$

where *C* is a positive constant depending only on *T*, *L*, and $\Vert \rho ^0\Vert _{\mathcal {W}^{1,\infty }_+}$. Indeed, we can similarly find from (A.1) that for $i =1,\cdots , d$

$$\begin{aligned} \begin{aligned} \frac{\partial }{\partial t}\partial _i \rho (t,F^{0,t}(x))&= -\partial _i u(t,F^{0,t}(x))\nabla \rho (t,F^{0,t}(x)) -\nabla \cdot u(t,F^{0,t}(x)) \partial _i \rho (t,F^{0,t}(x)) \\&\quad - \rho (t,F^{0,t}(x))\nabla \cdot \partial _i u(t,F^{0,t}(x)). \end{aligned}\end{aligned}$$

This implies

$$\begin{aligned} \begin{aligned} \Vert \nabla \rho (t,\cdot )\Vert _{L^\infty }&\le \Vert \nabla \rho _0\Vert _{L^\infty } \exp \left(C\int _0^t \Vert \nabla u(s,\cdot )\Vert _{L^\infty }\,ds \right) \\&\quad + \exp \left(C\int _0^t \Vert \nabla u(s,\cdot )\Vert _{L^\infty }\,ds \right)\int _0^t \Vert \rho (s,\cdot )\Vert _{L^\infty }\Vert D^2 u(s,\cdot )\Vert _{L^\infty }\, ds,\\&\le C\Vert \nabla \rho _0\Vert _{L^\infty } + C\int _0^t \Vert D^2 u(s,\cdot )\Vert _{L^\infty }\,ds \end{aligned}\end{aligned}$$

where we used $u \in \mathcal {W}^{1,\infty }(\mathbb R^d)$ and the estimate (A.4). On the other hand, $\Vert D^2 u(s,\cdot )\Vert _{L^\infty }$ can be estimated by

$$\begin{aligned} \Vert D^2 u(s,\cdot )\Vert _{L^\infty } \le \Vert \nabla W\Vert _{\mathcal {W}^{1,\infty }}\Vert \nabla \rho (s,\cdot )\Vert _{L^\infty }. \end{aligned}$$

Hence, we have

$$\begin{aligned} \Vert \nabla \rho (t,\cdot )\Vert _{L^\infty } \le C\Vert \nabla \rho _0\Vert _{L^\infty } + C\int _0^t \Vert \nabla \rho (s,\cdot )\Vert _{L^\infty }\,ds, \end{aligned}$$

and by applying Gronwall’s inequality to conclude the desired result. Similar arguments were used in [3] to construct classical solutions.

We next provide the a priori estimate of solutions to the system (1.1) in $\mathcal {W}^{1,1}_+(\mathbb R^d) \cap \mathcal {W}^{1,p}(\mathbb R^d)$. For notational simplicity, we set

$$\begin{aligned} {\widetilde{\mathcal {W}}}^{k,p}_+(\mathbb R^d) := \mathcal {W}^{k,1}_+(\mathbb R^d) \cap \mathcal {W}^{k,p}(\mathbb R^d) \quad \text{ for } \quad k \ge 0. \end{aligned}$$

### Proposition 10

Assume that the interaction potential *W* satisfies (5.1) for some $1 \le q \le \frac{d}{\alpha + 1}$. Let $\rho$ be the unique local-in-time solution to (1.1) constructed in [24] with initial data $\rho ^0$ satisfying $\rho ^0 \in (L^\infty \cap {{\widetilde{\mathcal {W}}}}_+^{1,p})(\mathbb R^d)$ where *p* is the Sobolev conjugate of *q*. Then there exists a $T^* > 0$ such that

$$\begin{aligned} \sup _{0 \le t \le T^*}\Vert \rho (t,\cdot )\Vert _{{{\widetilde{\mathcal {W}}}}^{1,p}_+} \le C, \end{aligned}$$

where *C* is a positive constant depending only on $T^*$, $\alpha$, *p*, and $\Vert \rho ^0\Vert _{{{\widetilde{\mathcal {W}}}}^{1,p}_+}$.

### Proof

The local-in-time well-posedness theory in [24] that

A.5 $$\begin{aligned} \frac{d}{dt}\Vert \rho \Vert _{{{\widetilde{\mathcal {W}}}}^{0,p}_+} \le C\Vert \rho \Vert _{{{\widetilde{\mathcal {W}}}}^{0,p}_+}^2. \end{aligned}$$

It also follows from (A.1)–(A.3) that

$$\begin{aligned} \frac{d}{dt}\Vert \nabla \rho \Vert _{L^1} \lesssim \Vert \rho \Vert _{{{\widetilde{\mathcal {W}}}}^{0,p}_+}\Vert \nabla \rho \Vert _{L^1} + \Vert \nabla \rho \Vert _{{{\widetilde{\mathcal {W}}}}^{0,p}_+} \le \Vert \rho \Vert _{{{\widetilde{\mathcal {W}}}}^{1,p}_+}^2, \end{aligned}$$

where we used $\Vert D^k u(t,x)\Vert _{L^\infty } \le C\Vert D^{k-1} \rho \Vert _{{{\widetilde{\mathcal {W}}}}^{0,p}_+}$ for $k \ge 1$ and $\Vert \rho \Vert _{{{\widetilde{\mathcal {W}}}}^{0,p}_+} \ge 1$. For the estimate of $\Vert \nabla \rho \Vert _{L^p}$, we obtain

$$\begin{aligned} \begin{aligned} \frac{d}{dt}\int _{\mathbb R^d} |\nabla \rho |^pdx&= -p\int _{\mathbb R^d} |\nabla \rho |^{p-2}\nabla \rho \cdot \left( D^2 \rho u + \nabla u \nabla \rho + \nabla (\nabla \cdot u) \rho + \nabla \cdot u \nabla \rho \right) dx\\&= (a) + (b) + (c) + (d), \end{aligned}\end{aligned}$$

where (*a*), (*b*), (*c*), and (*d*) are estimated as follows.

$$\begin{aligned} \begin{aligned} (a)&= -\int _{\mathbb R^d} u \cdot \nabla |\nabla \rho |^pdx = \int _\mathbb R\nabla \cdot u |\nabla \rho |^pdx \lesssim \Vert \rho \Vert _{{{\widetilde{\mathcal {W}}}}^{0,p}_+}\Vert \nabla \rho \Vert _{L^p}^p,\\(b)&\le p\int _{\mathbb R^d} |\nabla u| |\nabla \rho |^pdx \lesssim \Vert \rho \Vert _{{{\widetilde{\mathcal {W}}}}^{0,p}_+}\Vert \nabla \rho \Vert _{L^p}^p,\\(c)&\le p\Vert \nabla ^2 u\Vert _{L^\infty }\Vert \rho \Vert _{L^p}\Vert \nabla \rho \Vert _{L^p}^{p-1} \lesssim \Vert \nabla \rho \Vert _{{{\widetilde{\mathcal {W}}}}^{0,p}_+}\Vert \rho \Vert _{L^p}\Vert \nabla \rho \Vert _{L^p}^{p-1},\\(d)&\le p\int _{\mathbb R^d} |\nabla \cdot u| |\nabla \rho |^pdx \lesssim \Vert \rho \Vert _{{{\widetilde{\mathcal {W}}}}^{0,p}_+}\Vert \nabla \rho \Vert _{L^p}^p. \end{aligned}\end{aligned}$$

Thus, we get

A.6 $$\begin{aligned} \frac{d}{dt}\Vert \nabla \rho \Vert _{L^p} \le C\Vert \rho \Vert _{{{\widetilde{\mathcal {W}}}}^{1,p}_+}^2. \end{aligned}$$

Now, we combine (A.5) and (A.6) to deduce

$$\begin{aligned} \frac{d}{dt}\Vert \rho \Vert _{{{\widetilde{\mathcal {W}}}}^{1,p}_+} \le C\Vert \rho \Vert _{{{\widetilde{\mathcal {W}}}}^{1,p}_+}^2, \end{aligned}$$

and this concludes that there exists a $T^*>0$ such that

$$\begin{aligned} \sup _{0 \le t \le T}\Vert \rho (t,\cdot )\Vert _{{{\widetilde{\mathcal {W}}}}^{1,p}_+} \le C, \end{aligned}$$

where *C* is a positive constant depending only on $T^*$, $\alpha$, *p*, and $\Vert \rho ^0\Vert _{{{\widetilde{\mathcal {W}}}}^{1,p}_+}$. $\square$
